# Control over Banded Morphologies and Circular Dichroism in Chiral Halide Perovskites

**DOI:** 10.1021/acsnano.5c00472

**Published:** 2025-05-15

**Authors:** Matthew P. Hautzinger, Qiutong Ge, Md Azimul Haque, St. John Whittaker, Keisuke Yazawa, Stephanie S. Lee, Peter C. Sercel, Matthew C. Beard

**Affiliations:** † 53405 National Renewable Energy Laboratory, Golden, Colorado 80401, United States; ‡ Molecular Design Institute, Department of Chemistry, 5894 New York University, NY, New York 10003, United States; § Department of Metallurgical and Materials Engineering, Colorado School of Mines, Golden, Colorado 80401, United States; ∥ Center for Hybrid Organic Inorganic Semiconductors for Energy, Golden, Colorado 80401, United States; ⊥ Renewable and Sustainable Energy Institute, University of Colorado Boulder, Boulder, Colorado 80309, United States

**Keywords:** chirality, perovskites, spherulites, circular dichroism, thin films

## Abstract

Chiral halide perovskites (c-HPs) merge the chirality of organic cations with the semiconducting properties of metal halide frameworks, creating a family of chiral semiconductors with tunable chiroptoelectronic behavior. Here, we describe the impact of periodic banded morphologies of textured c-HP (*R/S*-NEA)_2_PbBr_4_ films (NEA = 1-(1-naphthyl)­ethylammonium) on their chiroptical behavior. Due to the interplay between the crystalline and glassy phases, the c-HP film growth is driven by rhythmic precipitation, producing a distinctive controllable radial banded pattern with the (*R/S*-NEA)_2_PbBr_4_ inorganic planes oriented parallel to the substrate. The banded morphology can be controlled, as evidenced by the growth temperature dictating the ridge-to-ridge spacing as well as the density of banded regions. The resulting circular dichroism (CD) spectral shape, intensity, and polarity vary in a seemingly random manner across processing conditions. However, these spectral features can be explained by considering key features of the banded morphology, such as refraction of the incident light due to surface morphology, birefringence, and stacked, rotated crystallites. These effects cannot be canceled by averaging front and back CD spectra of c-HP films, and our model incorporating these effects reproduces all observed CD spectra remarkably well. The control over the c-HP morphology and prediction capabilities of our CD modeling leads to further understanding of this class of semiconductors and the possibility of exploiting structural features for light polarization control akin to enhanced metamaterials.

## Graphical abstract

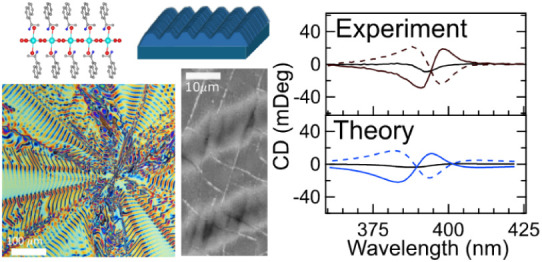

## Introduction

Hybrid organic–inorganic halide perovskites incorporating chiral organic cations are a promising class of chiral semiconductors, allowing for control over opto-spintronic phenomena.^1,2^ The chirality type and enantiomeric purity can be selected by the choice of the chiral organic cation that is incorporated into the structure while also maintaining the excellent semiconducting properties of the inorganic metal halide sublattice. Chiral halide perovskites (c-HPs) exhibit beneficial chiroptoelectronic behavior including strong circular dichroism (CD),^3–5^ circularly polarized photoluminescence,^6^ and chirality-induced spin selectivity.^7–11^ Understanding and elucidating the relationship of the chemical structure, composition, morphology, spin texture of the frontier bands, and so forth to the desired chiroptoelectronic properties are needed to advance this emerging class of chiral semiconductors.^1^

c-HP (*R/S*-NEA)_2_PbBr_4_ (NEA = 1-(1-naphthyl)­ethylammonium) (Figure 1a) is of particular interest because the structural chirality transfer from the chiral organic NEA cation to the Pb–Br inorganic framework causing helical distortions within the 2D sheets is well understood and fairly simple.^12^ This helical distortion is described by a screw axis along the inorganic planes of (*R/S*-NEA)_2_PbBr_4_ (*P*2_1_ space group). There already exist detailed crystal structures, hybrid-DFT simulations, and a semiempirical multiband k·p effective mass theory^13^ to describe the chiral excitonic electronic structure, including a detailed description explaining how the optical activity of the exciton arises, as well as how the CD is related to the spin textures of the frontier bands.^13–15^ In addition, (*R/S*-NEA)_2_PbBr_4_ has a low melting temperature and can form stable glasses via a facile quenching procedure.^16^ This promotes a unique crystallization process that begins with an amorphous composition and proceeds with subsequent crystallization, i.e., melt processing, as demonstrated with select halide perovskite compounds.^17–23^ The interesting crystallization kinetics of (*R/S*-NEA)_2_PbBr_4_ can be exploited for textured or unique growth, as previously observed in other halide perovskites.^24^ Therefore, this system represents a prototypical member of the c-HP family of chiral semiconductors that can be used to develop the needed intuition for controlling their chiroptoelectronic behavior.

**Figure 1:**
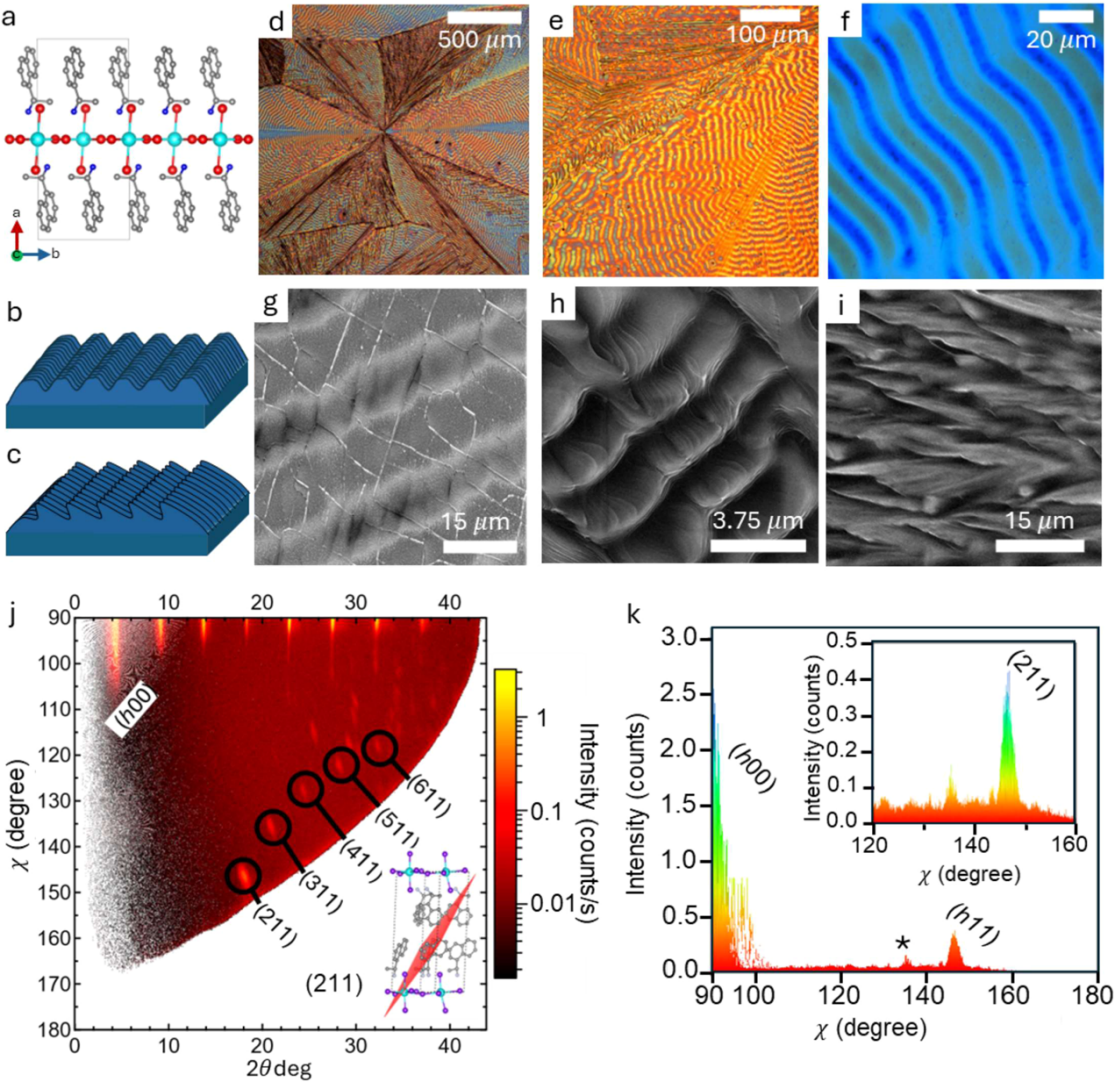
Textured film growth of (*R/S* **-**NEA)_2_PbBr_4_. (a) Crystal packing diagram of (*S*-NEA)_2_PbBr_4_ (atom colors: Pb = teal, Br = red, C = gray, N = blue; H atoms omitted for clarity). Schematic of textured films with (b) ridges and (c) dunes. (d–f) Optical microscope images of film growth highlighting the multiple crystal orientations in different sectors (140 °C anneal, 100 mg/mL concentration). SEM images of (g) DMF-based growth highlighting the valleys vs flat top ridges. (h) Dune-like morphology of the 2-ME based growth. (i) Fibril growth that intersperses different sectors of the film (representative for both DMF and 2-ME). More SEM images are shown in Figure S3. (j) XRD collected with a 2D detector transformed to a 2D χ vs 2θ plot. The (*h*00) planes dominate the χ = 90° axis, indicating the orientation of the inorganic planes parallel to the substrate. Side reflections with the largest intensity can be indexed to the (*h*11) family of planes. (k) shows the low intensity of the (211) plane relative to the (*h*00) family, and * shows the low intensity of the unassigned peaks, indicating very few off-axis crystallites are present.

For systems that undergo a glass-to-crystal phase transition, the slow diffusion rate (relative to the crystallization rate) in the viscous glass can lead to periodic oscillations in the thickness and strain at the crystal growth front/interface. This periodic banding is described by a rhythmic precipitation where there is a periodic “wrinkling” of the resulting film, with ridgelines and valleys of thicker and thinner films.^25–27^ These structures are referred to as banded spherulites, where there is radial growth from a point nucleation and periodic concentric banded rings throughout the film as seen under optical microscopy.^28^ The observed concentric rings of bands can be due to changes in the thickness, or twisting at the crystal growth interface, with crystallites lying edge-on (i.e., orthogonal to the substrate) and flat-on (parallel to the substrate). The oscillation between these two orientations about the growth direction results in concentric interference bands when viewed between crossed polarizers due to orientation-dependent refractive indices.^29,30^ Spherulites not only are an interesting growth habit but also have implications in modulating light polarization for a variety of applications.^31^

Here, we describe a unique banded spherulite morphology in (*R/S*-NEA)_2_PbBr_4_ thin films and their influence on chiroptical properties. Microscopy, atomic force microscopy (AFM), and X-ray diffraction (XRD) characterization suggest that these banded structures result from rhythmic precipitation dynamics, where alternating thicker and thinner regions form the bands, rather than twisted structures. Depending on the solvent choice, the bands can possess either a ridge/valley morphology (Figure 1b) or a dune-like pattern (Figure 1c). In addition, we demonstrate that the growth temperature can control the ridge-to-ridge spacing as well as the density of banded regions in the film. The CD response of these films is anomalous, with polarity and spectral shape varying based on the deposition conditions. By modeling factors such as incident light angles, strain-induced relative birefringence, and twisting akin to stacking faults, we successfully replicated the seemingly anomalous CD behavior, offering a framework to understand and predict these effects.

## Results and Discussion

Films of (*R/S*-NEA)_2_PbBr_4_ were processed by spin coating from either *N*,*N*-dimethylformamide (DMF) or 2-methoxyethanol (2-ME) and subsequent annealing and crystallization of (*R/S*-NEA)_2_PbBr_4_. As can be seen under optical microscopy (in reflection mode and without polarization), banded spherulite morphologies are observed, with domains of fibrils interspersed in between (Figure 1d). A characteristic Maltese cross is present in an (*R*-NEA)_2_PbBr_4_ spherulite imaged between crossed polarizers, confirming that crystalline fibers are oriented radially with respect to the spherulite center (Figure S1).^28^ Insertion of a full-wave plate further revealed that the large refractive index is along the radial growth direction (i.e., the spherulite is positive; Figure S1). Banding of varying periodicity can be observed within the same film in different sectors (Figure 1e), although the periodicity is highly uniform within a single sector (Figure 1f). In scanning electron microscopy (SEM) images, there appear to be three unique domains. First, there is the flattened ridge-valley case (Figure 1b) that was produced when processing from DMF (Figure 1g). In this domain, it appears under SEM that there are flat crystal growth (ridge) regions followed by periodic dips (valley) in the film. AFM height maps confirm the presence of periodic height variations in the film exhibiting banding (Figure S2). There appears to be cracking on the surface, visible only under SEM, which could be due to contraction during the growth or cooling process. When processed from the more volatile solvent (i.e., faster evaporating) 2-ME, a morphology of dune-like fields is formed (Figure 1h). These films grown with 2-ME possess highly periodic asymmetric dune-like features with sharp cutoffs. Distinct crystallites can be observed where there are flat 2D crystal domains stacked upon each other, but no edge-on crystal domains are observed, indicating no twisting of the crystallites relative to each other. The other motif observed interspersed between the periodic band structures is a branched fibrillar morphology that is observed in both DMF and 2-ME films (Figure 1i). Here, fibrils grow radially outward. Striations within the fibrils are present, but these regions do not appear to have significant edge-on oriented crystal domains. In highly selective sampling under SEM, seemingly twisted fibrils were observed, though these were few and far between and not indicative of the general growth habit (Figure S3d).

XRD of thin films processed at a variety of temperatures with both DMF and 2-ME demonstrates that the films are oriented with the inorganic planes of (*R/S*-NEA)_2_PbBr_4_ parallel to the substrate (Figure S4). In films with the 2D inorganic planes lying parallel to the substrate, we expect diffraction peaks exclusively of the (*h*00) family (note that the *a*-axis is orthogonal to the inorganic planes in (*R/S*-NEA)_2_PbBr_4_ (Figure 1a)). The absence of (011) or (111) peaks indicates no contribution of edge-on crystallites in the diffraction pattern.^32^

2D X-ray diffraction patterns collected on the films revealed that the inorganic lattice is almost completely parallel to the substrate (Figure 1j). The only off-axis diffraction peaks (i.e., χ ≠ 90°) of reasonable intensity can be assigned to the (*h*11) family of planes, with (211) only having modest intensity compared to the (*h*00) planes. Note that the χ = 90° configuration represents the substrate surface’s normal direction. At these off-normal χ angles, the (*h*11) planes should appear when the (*h*00) plane is preferentially oriented parallel to the substrate, which further indicates the banded morphology contains only one preferred crystal orientation. There are some extremely low-intensity diffraction off-axis peaks that we could not assign to specific diffraction planes. These possibly originate from unoriented (i.e., nonparallel) crystal domains, as seen with the fibrils that exist in the film where twisting or off-axis crystallites occur. The low intensity of these diffraction peaks indicates that they are an extreme minority (Figure 1k). The diffraction patterns from films grown at different temperatures show the same (*h*00) family of planes, indicating no change in the texturing of the films (Figure S4). There is, however, a large increase in the intensity of the diffraction peaks as the temperature increases. This suggests that the higher temperature annealing with more banding morphology is more crystalline with less amorphous content, leading to larger diffraction intensities. This is corroborated by a recent work with X-ray nanoprobe measurements on the same system.^33^

We investigated processing control to observe changes in the growth behavior. Figure 2a–f shows the film morphology of (*S*-NEA)_2_PbBr_4_ spin-coated from DMF and annealed at varying temperatures from 110 to 160 °C. From 110 to 150 °C, there appears a trend that the amount of banded morphology increases relative to fibril/branched morphologies, i.e., there is more banding at higher temperatures. The branching being more prevalent at lower temperatures indicates that the thermodynamic growth habit is more likely to form as branched fibrils. At elevated temperatures, the nucleation and growth as a banded film are likely promoted by a kinetic process. The growth rate was not quantified, but full coverage of the substrate with crystallites requires 20 min at 110 °C and is complete in <5 min at 160 °C. At 160 °C, the thin film is dominated by textured domains with sparse regions of degraded/ablated film interceded, and at 170 °C, the film is almost completely ablated. The periodic spacing of the bands in these morphologies is dependent on growth temperature. The ridge-to-ridge distance can be tuned from <5 μm (110 °C) to >30 μm (160 °C) and increases with growth temperature (Figure 3a), though there is some variability within individual samples of band-to-band distance in separate crystallites. The increase in the band spacing is a result of the growth rate of the crystalline domains. Corresponding optical micrographs collected between crossed polarizers are displayed in Figure S8.

**Figure 2:**
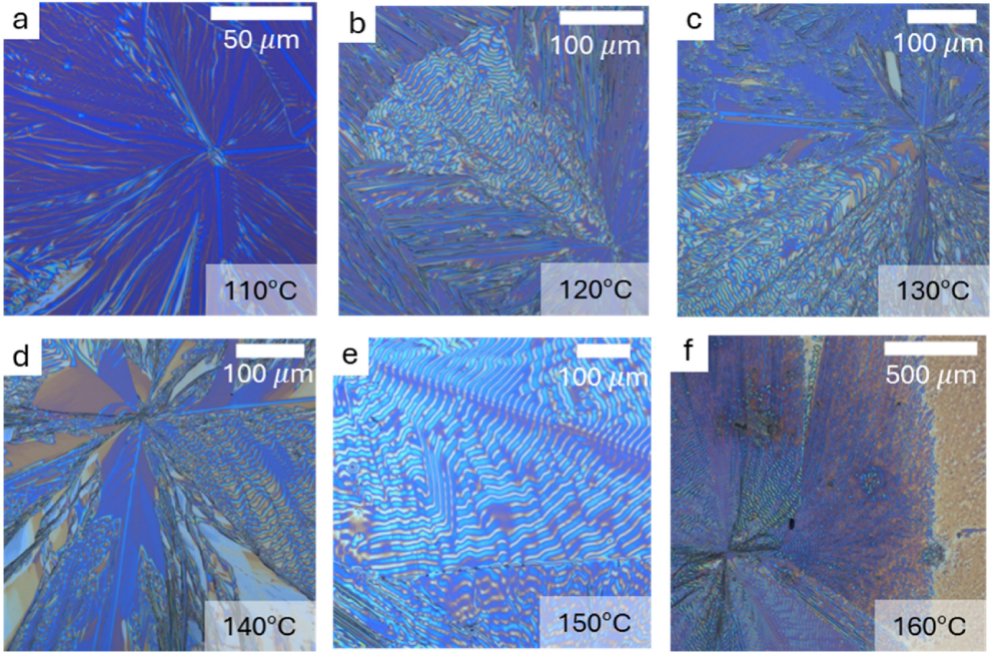
(a–f) Optical microscopy of (*S*-NEA)_2_PbBr_4_ films grown from DMF at temperatures from 110 to 160 °C. Further microscopy on the other conditions/enantiomers is shown in Figures S5–S7. The general trend is that crystallites have more fibril domains at low temperatures, with increasing regions of banding at elevated temperatures. At 160 °C, the film begins to slightly degrade as can be observed in panel f.

**Figure 3:**
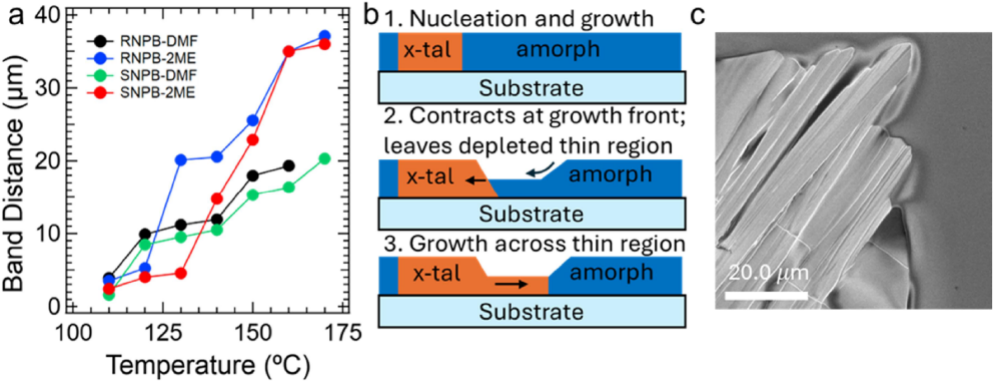
Observations about the banded growth. (a) Increase of ridge-to-ridge distance (band distance) with annealing temperature formed by the rhythmic precipitation. (b) Proposed mechanism for the growth of the periodic oscillations in film thickness. Initially, there is nucleation taking place in the amorphous precursor (from spin coating or a preformed glass). Simultaneously, there is an effective contraction of the total material on the substrate due to the higher density of the crystalline phase relative to the amorphous. This leads to a depleted thin region of the precursor, in which the crystallite continues to grow through producing the “valleys” until it reaches the thick portion of amorphous material again. (c) shows the proposed amorphous growth front.

A proposed mechanism for the formation of this banded morphology is as follows: as the crystallites grow, there is contraction at the crystal growth front interface with the viscous amorphous precursor (Figure 3b). This contraction is due to the higher density of the crystallites relative to the amorphous precursor, which creates a thinner depleted region of amorphous precursor material at the growth front. The amorphous material (Figure 3c) is likely similar to the glass phases observed in these systems^16^ and has extremely sluggish diffusion across the substrate, which does not allow for replenishment at the crystal growth front. Interestingly, other halide perovskites also can form a similar but less periodic wrinkled texture during thin-film crystallization,^34,35^ which can be induced by cold antisolvent and substrate conditions, supporting that texturing can be induced by viscous precursor material.^24^ To support the importance of the amorphous/glassy phase, racemic (NEA)_2_PbBr_4_ does not form a glassy phase^16^ and we did not observe periodic banding during thin-film deposition (Figure S9). In addition, the amorphous/glass and crystallite interface can be observed clearly under SEM in Figure 3c.

Texturing and morphology can influence the CD response and produce effects such as apparent CD, particularly in low-dimensional perovskite films.^36^ Figure 4 and Figures S10–S13 show CD spectra of (rac/*R/S*-NEA)_2_PbBr_4_ films grown with different annealing temperatures and solvents. Although there were trends in morphological changes with temperature, as described above, we were unable to discern a systematic trend in the changes to the CD spectra. None of the films reproduce the shape of CD spectra inferred from reflectivity measurements of single crystals of (*R/S*-NEA)_2_PbBr_4_,^15^ which show that the excitonic CD spectra exhibit a bisignate response (Cotton effect) with negative polarity in the higher energy feature for (*S*-NEA)_2_PbBr_4_. The seemingly random variations in CD features suggest that they may be describable in terms of “apparent CD” or the linear dichroism linear birefringence (LDLB) effect, wherein there is a misalignment between the linear dichroism and the linear birefringence of the sample.^37–39^ For excitonic apparent CD specifically, Salij et al.^37^ have shown that this effect can occur if the exciton electronic structure possesses nondegenerate levels whose electric transition dipoles are nonorthogonal and not parallel.^37^ Here, we evaluate whether a simple LDLB approach can explain the observed variations. A common procedure to determine if apparent CD is present is to collect CD from the front and back sides of the films, i.e., flipping the films and remeasuring the CD. Apparent CD resulting from coupling of LB and LD will appear as a monosignate peak that is antisymmetric with the reversal of the direction of light, assuming frontside/backside symmetry in the morphology of the sample.^37^ The first obvious CD feature in Figure 4 is that *all* of the spectra are nonsymmetric with respect to measurement from the front side versus the back (substrate) side (Figure 4a solid red traces compared to dashed red traces). While there may be an apparent CD effect, the lack of front/back symmetry in these spectra is not surprising since the film texture is not generally symmetric between the front side and the back (substrate) side. Moreover, some of these spectra show monosignate CD spectra when measured only from one direction, while the opposite is not the case, or when averaging both from the front and the back (e.g., Figure 4, 140 °C). The averaged front and back spectra of some samples appear monosignate, whereas the intrinsic CD as modeled by us and supported by the literature^14^ should be bisignate (Figure S2-1). This is in contrast to the common assumption that apparent CD effects in thin films can be eliminated by averaging the CD spectra measured from both the front and back sides.^36^

**Figure 4:**
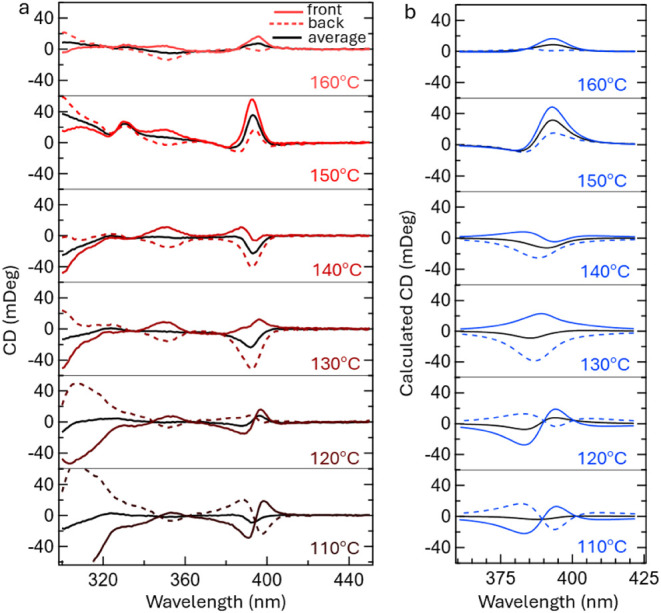
Circular dichroism (CD) of (*S* **-**NEA)_2_PbBr_4_ films processed from DMF at increasing annealing temperatures and modeling of the CD. Figures S10–S13 show the CD of the other conditions/enantiomers. (a) CD spectra of samples from 110 to 160 °C. (b) Calculated models of films with varying relative birefringence (δ), twist (γ), and refraction as outlined in Table 1. A detailed discussion of these effects is outlined in the Supporting Information.

Furthermore, there are certain CD spectra that are particularly puzzling. For example, the CD spectra of films processed at 110 °C are bisignate. At first glance, these spectra appear to exhibit the Cotton effect, as expected for intrinsic excitonic CD. However, excitonic intrinsic CD is symmetric with respect to direction reversal, while these spectra are *antisymmetric* with respect to measurement on the front and back sides (equivalent to reversal of the direction of light propagation relative to the sample normal). Another form of apparent CD can occur for double-layered structures with a relative twist about the optical axis.^37^ However, this phenomenon is symmetric with respect to direction reversal, while the measured spectra here are antisymmetric. Therefore, all the measured CD appear to be anomalous with little hope of understanding the intrinsic response or of tailoring a particular chiroptical behavior *a priori*. However, we can reproduce all of these features (Figure 4b) by accounting for a few morphological features found within the films.

In Supporting Note S2, we describe the crystal structure and an exciton fine structure model for chiral (*R/S*-NEA)_2_PbBr_4_ based on the effective mass approximation as well as an extensive discussion describing textures and their effect on CD. Here, we will highlight a few dominant mechanisms used to reproduce Figure 4 CD spectra. While no individual mechanism explains all the CD features observed, the combination of multiple morphology effects (Table 1) can reproduce the spectra. This is likely related to the fact that our CD measurements sample an ensemble across multiple domains. One mechanism we uncovered for generating CD was to twist the structures of two domains of (*R/S*-NEA)_2_PbBr_4_ relative to each other (Figure 5a) at angle γ similar to previous reports.^39,40^ This produces a symmetric CD response (i.e., the same front and back) depending on the angle of twist (Figure 5b) which does not explain the observed antisymmetric CD. However, a minor twist of 2° (Figure 5c) produces a large CD response and can be combined with other effects, described below, to produce part of the spectral shape (e.g., Figure 4a, 120 °C). Another proposed effect is that the banded structures and edges of fibrils in the textured morphology will cause light to refract and propagate at an angle θ_mat_ off normal to the plane of the inorganic layers (Figure 5d). This refraction allows the electric field vector of the light to couple to components of the electric dipole transition vectors not accessible with light that is normally incident on the inorganic layers and allows for a CD response even in nonchiral media (more in S2-4-2-1). Figure 5e shows the dependence of the peak CD on the refraction angle, producing an asymmetric CD response with respect to the refraction angle, while Figure 5f shows that the calculated CD spectra for front-side illumination is monosignate much like many of the experimental CD spectra measured from the front or back side. However, based on the response of front/back simulations of the ridged structure for a CD response for ridge films with a flat backside (Figure S2–12), the response is somewhat diminished, particularly when considering the front/back measurement. This motivates a secondary effect distinct from that of refraction.

**Table 1:** Parameters for Calculated CD Spectra Given in Figure 4b

Film temp. (°C)	Ridge Azimuth (ϕ)	Relative Birefringence (δ)	Twist (γ)	Front (θ_mat_)	Back (θ_mat_)
160	–45°	0.0005	0.017°	±5°	±0°
150	–45°	0.0005	–0.5°	±6.5°	±0°
140	45°	0.001	0°	±5°	±0°
130	45°	0.0015	–0.2°	±4°	±0°
120	–45°	0.002	–0.3°	±7°	±7°
110	–45°	0.002	0°	±7°	±7.5°

**Figure 5:**
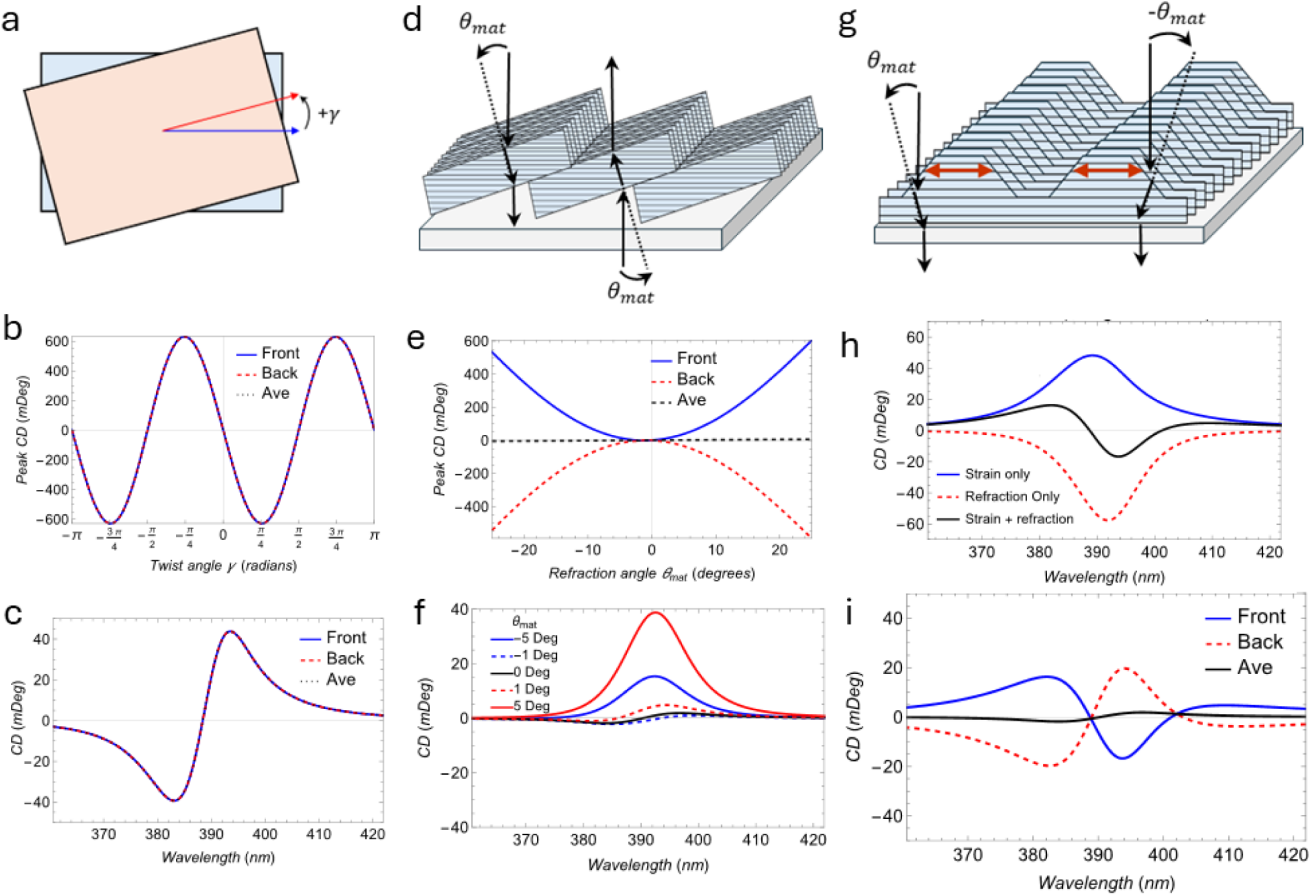
Types of texture that produce apparent CD. (a) Schematic of the twist between two domains of (*R/S*-NEA)_2_PbBr_4_ at angle γ. (b) Dependence of peak CD on γ angle with a symmetric response and large magnitudes. (c) Calculated CD spectra for a −2° twist. (d) Schematic of the refraction angle (θ_mat_). (e) Dependence of peak CD on θ_mat_ for a dune aligned at an azimuth angle ϕ = *–*45° to the *a*_*1*_ axis showing the asymmetric response with respect to refraction angle. (f) Calculated CD spectra from the frontside for ridges for various values of θ_mat_ at azimuth angle ϕ = −45°. (g) Schematic of the relative birefringence (red lines) between layers with refraction effects incorporated. (h) Calculated front-side CD spectra showing the effect of refraction only, relative birefringence only, and the combination of the two to produce a bisignate response. (i) Calculated CD spectra from relative birefringence and refraction effects from the front and backside.

In addition to refraction, the ridge and dune structures likely have their birefringence major axis aligned along the radial axis of growth (indicated in Figure 2a), where the contrast for ridges aligned parallel to the polarizer is lower in comparison to those that are misaligned. This is further supported by the images in Figure S1 where the Maltese cross-like pattern indicates an aligned radial axis of growth. If we suppose that different heights in the films have varying strain, this birefringence can be added into the model as a relative birefringence (δ) between the ridge structure and the bottom of the film (described in S2-4-3). This relative birefringence in the ridges produces a monosignate and asymmetric response (Figure S2-14, Figure S2-15). The most realistic situation is that both refraction and relative birefringence occur simultaneously (Figure 5g). When both refraction effects and relative birefringence are present, the two responses are slightly shifted spectrally and can possess opposite polarity (Figure 5h). Consequently, when both effects are present, a bisignate apparent-CD response that is antisymmetric with respect to front vs back side measurement can be observed (Figure 5i). Coexistence of the various effects can be used to explain the variation of the observed CD responses (Figure 4). Table 1 shows the combinations of these features used to reproduce each spectrum with varying amounts of refraction, relative birefringence, and twisting. One observation and possible trend is that the relative birefringence (possibly produced by strain) parameter decreases with increasing temperature. We find this result insightful, as we expect higher-temperature annealing to produce a less strained sample.

We have, to the best of our ability, captured a suite of morphological features within these thin films, though limitations remain such as incorporating the amount of amorphous content, extending to capture more morphological features such as the fibril morphology, which is only lightly discussed, as well as further spatial mapping of the CD to see each feature as an ensemble and direct our modeling. The model provided in this system, based on an analytical effective mass description, can be applied to other excitonic crystalline semiconductors with textured morphologies. The key, as mentioned, is understanding the morphology with a focus on film microstructure, e.g., strain, and the relative orientation of crystallites to each other and to incident light. We expect that this modeling framework can be utilized in future systems, for example, in intentionally textured thin-film systems or metamaterials, to optimize control of light polarization.

## Conclusions

The textured films of (*R/S*-NEA)_2_PbBr_4_ show a controllable morphology, which changes the banded spherulites’ ridge-to-ridge distance as well as the distribution of various morphological features. Our microscopy and diffraction experiments demonstrate that the growth produces films with the inorganic planes of (*R/S*-NEA)_2_PbBr_4_ lying parallel to the substrate. These unique textures provide rich features in the CD spectra, with the shape, polarity, and magnitude changing with the morphology. By introducing a model based on twisting, refraction, and relative birefringence, we can explain these anomalous CD spectra in greater depth than LDLB effects as done previously. We believe that our demonstrated understanding of these effects can be exploited to produce controllable CD in films of chiral hybrid perovskites, which provides opportunities for advancing semiconducting metamaterials for chiroptoelectronic property control.

## Materials and Methods

### Materials

Lead­(II) bromide, hydrobromic acid (48 wt %), *R/S*-(−)-1-(1-naphthyl)­ethylamine, diethyl ether, DMF, 2-Me, isopropyl alcohol, chlorobenzene, and poly­(9,9-dioctylfluorene-alt-*N*-(4-s-butylphenyl)-diphenylamine were purchased from Sigma-Aldrich.

### (*R/S*-NEA)_2_PbBr_4_ Single Crystal Growth

Lead­(II) bromide (90.0 mg, 0.245 mmol) was dissolved in 1.0 mL of concentrated hydrobromic acid by heating at 85 °C. After the solution was cooled to room temperature, 2.4 mL of DI water was slowly added to the mixture. Subsequently, the solution was cooled to 0 °C, and *R/S*-(1-naphthyl)­ethylamine (87 μL, 0.48 mmol) was added dropwise. This mixture was then heated at 95 °C while stirring for a minimum of 2 h to ensure complete dissolution of precipitates. The solution was then left to cool to room temperature at a rate of 2 °C/h, and crystals were collected over vacuum filtration.

### Thin-Film Growth

Spin-coating depositions were performed in a nitrogen environment. Substrates (glass or silicon wafer) were prepared by sonicating in isopropanol (15 min) followed by UV-Ozone treatment (15 min). Precursor solutions were prepared by dissolving (*R/S*-NEA)_2_PbBr_4_ crystals in DMF at 200 mg/mL, followed by filtration with a 0.22 μm PTFE filter. Chosen concentrations were prepared by serial dilutions from this stock solution. The solution was spin-coated for 20 s at 4000 rpm, followed by immediate annealing at specified temperatures. Specifically, for the circular dichroism (Figures 4, S10, and S11) and X-ray diffraction (Figures 1, S1, and S2), films were annealed as follows: 110 °C for 20 min, 120 °C for 15 min, 130 °C for 10 min, 140 °C for 5 min, 150 °C for 4 min, and 160 °C for 3 min. Modifications to this growth are noted in the text. Annealing for extended times at high temperatures (≥150 °C) resulted in a loss of material on the substrate.

#### Microscopy

An Axio Imager.M2m optical microscope was used for optical images, except for Figures S1 and S8 where an Olympus BX53 microscope equipped with a polarizer, an analyzer, and a first-order tint plate was used for viewing the samples. Micrographs were recorded with an Olympus DP74 camera. A Hitachi S-4800 scanning electron microscope was used for SEM micrographs. SEM images were collected using a Hitachi S-4800 scanning electron microscope. A conductive layer of TFB [(poly­(9,9-dioctylfluorene-alt-*N*-(4-s-butylphenyl)-diphenylamine)] was spin coated on top of samples (10 mg/mL TFB in chlorobenzene, spin coating at 4000 rpm, 20 s, followed by 2 min of annealing at 100 °C). This enhanced the image quality compared to uncoated samples or samples coated with 5 nm of Pt (sputtered).

#### Spectroscopy

The absorbance spectra of the films were measured using ultraviolet–visible spectroscopy (Cary 6000i). CD measurements were carried out using an Olis DSM 170 spectropolarimeter at 1 nm intervals with 1 s integration time. When rotating the sample between the front and back, the CD spectra were collected at the same position on the film.

#### Diffraction

Powder X-ray diffraction (XRD) data were recorded using a Bruker D8 Discover X-ray diffractometer with a Hi-Star 2D area detector using Cu Kα radiation (1.54 Å).

#### Modeling

A symmetry-informed model for the exciton fine structure in chiral (*R/S*-NEA)_2_PbBr_4_ was developed using multiband effective mass theory. The exciton fine structure model is used to compute the complex dielectric response of the material, with corrections for magnetic dipole transitions, and strain, if present. To compute the optical properties, we insert the tensor dielectric function into the wave equation and solve for the normal modes for light propagation for a given incidence direction with respect to the crystal axes. CD calculations exploit biorthogonality relations to perform normal mode decomposition (an essential procedure since the dielectric tensor is complex and is neither Hermitian nor symmetric, and the normal modes, which are elliptically polarized, are not orthonormal in the usual sense). This approach, applied to textured and/or multilayered films, captures both intrinsic and “apparent” CD effects; see Supporting Note S2 for details.

## Supplementary Material



## Notes

Materials and methods and extra figures, cross-polarized microscopy, AFM data, further SEM characterization, further XRD characterization, optical microscopy of other enantiomers, CD of other enantiomers, and absorbance spectra (Section 1); supporting note on the CD/apparent CD model including background, exciton fine structure model for (*R/S*-NEA)_2_PbBr_4_, and models for intrinsic CD, apparent CD, and extrinsic CD in planar and nonplanar films (Section 2) (PDF)
