# Supplementary material for: Control over Banded Morphologies and Circular Dichroism in Chiral Halide Perovskites
Source: ACS Nano. 2025 May 15;19(20):19141–8. doi: 10.1021/acsnano.5c00472 (PMC12120980; doi:10.1021/acsnano.5c00472)
Supplement: Supplementary file 1 [file nn5c00472_si_001.pdf]

Supporting Information for

Control over Banded Morphologies and Circular Dichroism in Chiral Halide Perovskites

Matthew P. Hautzinger,[1]* Qiutong Ge,[1] Md Azimul Haque,[1] St. John Whittaker,[2] Keisuke Yazawa,[1,3] Stephanie S. Lee,[2] Peter C. Sercel,[4] Matthew C. Beard[1,5]*

[1]National Renewable Energy Laboratory, Golden, Colorado 80401, United States

[2]Molecular Design Institute, Department of Chemistry, New York University, New York, NY 10003, USA

[3]Department of Metallurgical and Materials Engineering, Colorado School of Mines, Golden, Colorado 80401, USA

[4]Center for Hybrid Organic Inorganic Semiconductors for Energy, Golden, CO 80401, USA

[5]Renewable and Sustainable Energy Institute, University of Colorado Boulder, Boulder, CO 80309, USA

Matthew C. Beard – *Email:* matt.beard@nrel.gov

Matthew P. Hautzinger – *Email:* matthew.hautzinger@nrel.gov

Contents

S1. Figures of further characterization 4
Figure S1. Cross-polarized microscopy of (R-NEA)$_2$PbBr$_4$ 4
Figure S2. AFM data 4
Figure S3. Additional SEM images of the banded structures. 5
Figure S4. XRD of (S-NEA)$_2$PbBr$_4$ 6
Figure S5. (a-f) Optical microscopy of (S-NEA)$_2$PbBr$_4$ films grown from 2-ME 6
Figure S6. (a-f) Optical microscopy of (R-NEA)$_2$PbBr$_4$ films grown from DMF at temperatures from 110-160°C. 7
Figure S7. (a-e) Optical microscopy of (R-NEA)$_2$PbBr$_4$ films grown from 2-ME 7
Figure S8. Cross polarized microscopy 8
Figure S9. (a-c) Optical images of a spin coated film of racemic compound (*rac*-NEA)$_2$PbBr$_4$. 8
Figure S10. (a) Circular dichroism and (b) absorbance of (*rac*-NEA)$_2$PbBr$_4$. 9
Figure S11. CD of (S-NEA)$_2$PbBr$_4$ processed from 2-ME 10
Figure S12. CD of (R-NEA)2PbBr4 processed from DMF 11
Figure S13. CD of (R-NEA)2PbBr4 processed from 2-ME 11
Figure S14. Absorbance of (S-NEA)$_2$PbBr$_4$ 12
Figure S15. Absorbance of (R-NEA)$_2$PbBr$_4$ 12
S2. Supplementary note on CD/apparent-CD model 13
S2-1. Introduction 13
S2-2. Crystal structure and exciton fine structure model for chiral *S/R* NPB 13
S2-2-1. Crystal structure for chiral *S/R* NPB 14
S2-2-2. Exciton fine structure in chiral *S/R* NPB 14
S2-3. Model for intrinsic circular dichroism, planar films 16
S2-4. Models for circular dichroism in non-planar films 18
S2-4-1. Interlayer twist 18
S2-4-2. Refraction effects. 20
S2-4-2-1. Refraction effects: Dune morphology. 23
3D extrinsic CD effect. 24
Apparent CD effect for dunes along azimuths ±45 *degrees.* 26
S2-4-2-2. Refraction effects: Ridge morphology. 27
S2-4-3. Ridge birefringence effects. 28
S2-4-3-1. Ridge birefringence effects with refraction. 31

References 32

List of Supplementary Tables

Table S1. Crystal structure and local formal dipole moments in *S*-NPB 14
Table S2. Electric & magnetic dipole transition matrix elements, point group C_2 15
Table S3. Comparison of fine structure in *S*- and *R*-NPB 16
Table S4. Transition dipoles for *S*-NPB in rotated coordinates 22

List of Figures pertaining to CD modelling (Supplementary note 2)

Figure S2- 1. CD spectra for planar films of chiral *S/R* NPB 18
Figure S2- 2. Exciton CD response of two stacked grains. 19
Figure S2- 3. Exciton CD response of three stacked grains. 19
Figure S2- 4. Model depiction of refraction in films with dune- and ridge-like morphology. 20
Figure S2- 5. Model depiction of coordinates to describe refraction in films with dune- and ridge-like morphology. 21
Figure S2- 6. Apparent CD response for dunes. 23
Figure S2- 7. Apparent CD response for dunes at dune azimuth ϕ=0 degree. 24
Figure S2- 8. 3D extrinsic CD response for dunes at dune azimuth ϕ=90 degree. 25
Figure S2- 9. 3D extrinsic CD response for dunes at dune azimuth ϕ=90 degree, symmetry C_{2v} and D_{2h}. 25
Figure S2- 10. Apparent CD response for dunes at fixed azimuths $\phi = \pm 45$ degrees. 26
Figure S2- 11. Apparent CD response for symmetric ridges. 27
Figure S2- 12. Apparent CD response for ridged films with flat backside. 28
Figure S2- 13. Cartoon depicting birefringence aligned to ridges. 29
Figure S2- 14. Apparent CD response for ridged films with uniform birefringence (no refraction)....... 30
Figure S2- 15. Apparent CD response for ridged films with birefringence in the ridge top (no refraction). 31
Figure S2- 16. Apparent CD response for ridged films with birefringence and sidewall refraction. 32

S1. Figures of further characterization.

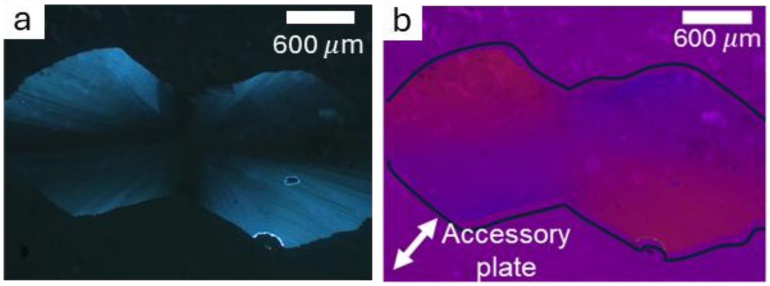

Figure S1. Cross-polarized microscopy of (R-NEA)$_2$PbBr$_4$ showing (a) Maltese cross under a polarizer across a single spherulite. (b) The same crystallite as panel (a) showing cross-polarized micrograph of (R-NEA)$_2$PbBr$_4$ with an Olympus U-TP530 first order tint plate.

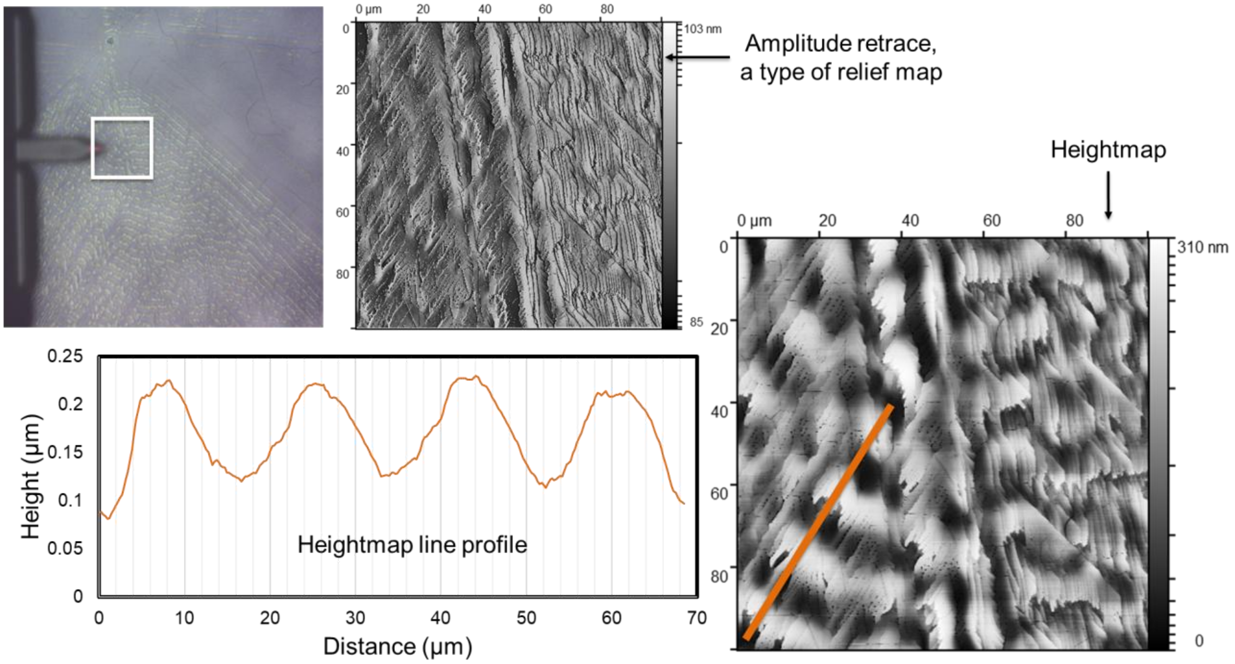

Figure S2. AFM data of (R/S-NEA)$_2$PbBr$_4$ films.

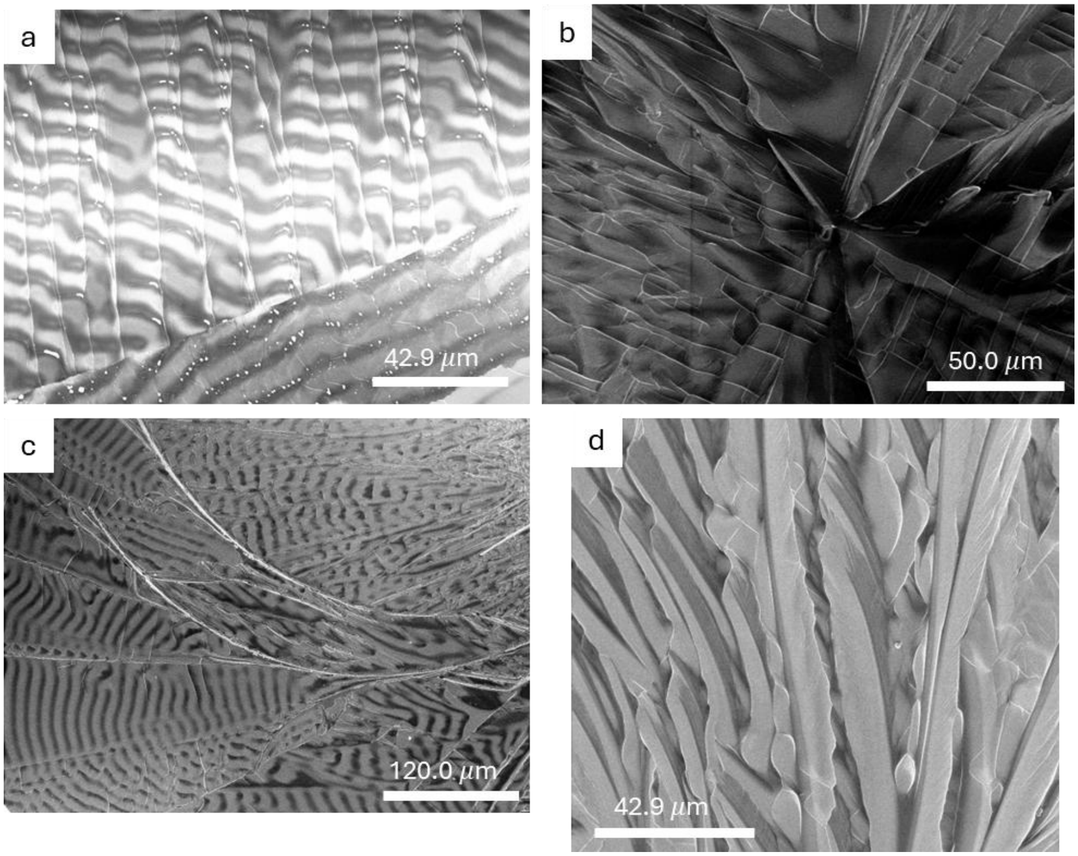

Figure S3. Additional SEM images of the banded structures. (a) Clear banding shown under SEM. (b) The core of one of the banded structures. (c) Mixed fibril banded region of the morphology. (d) A sparsely found twisted structure.

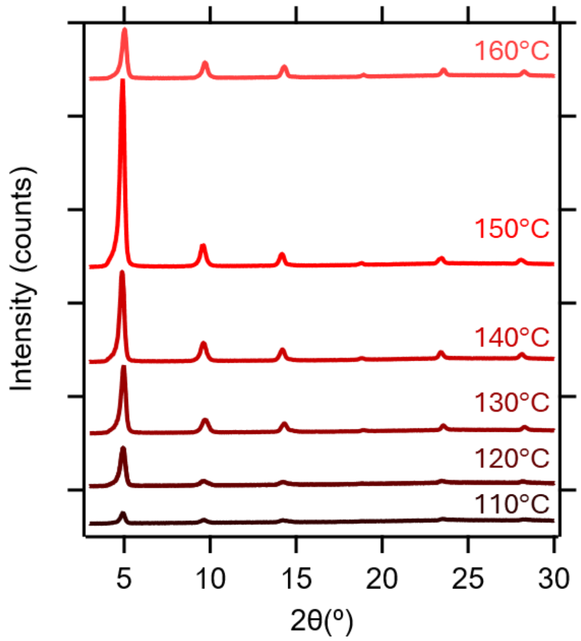

Figure S4. XRD of (S-NEA)$_2$PbBr$_4$ processed at varying temperatures as noted on the plot.

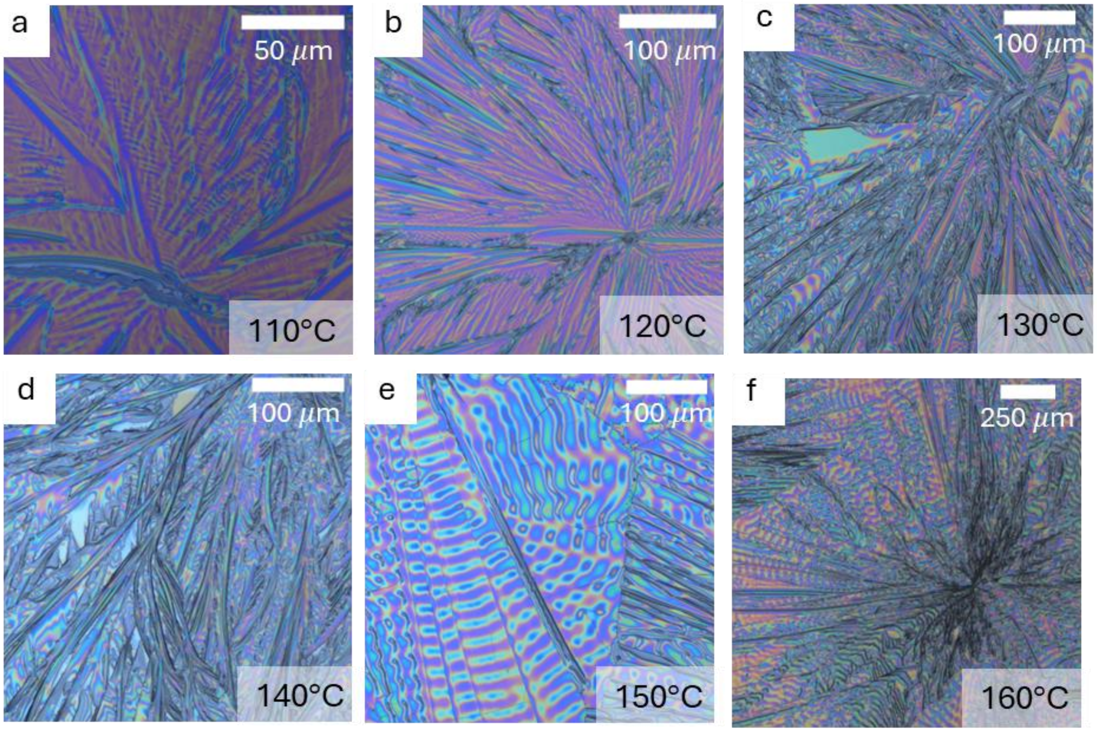

Figure S5. (a-f) Optical microscopy of (S-NEA)$_2$PbBr$_4$ films grown from 2-ME at temperatures from 110-160°C.

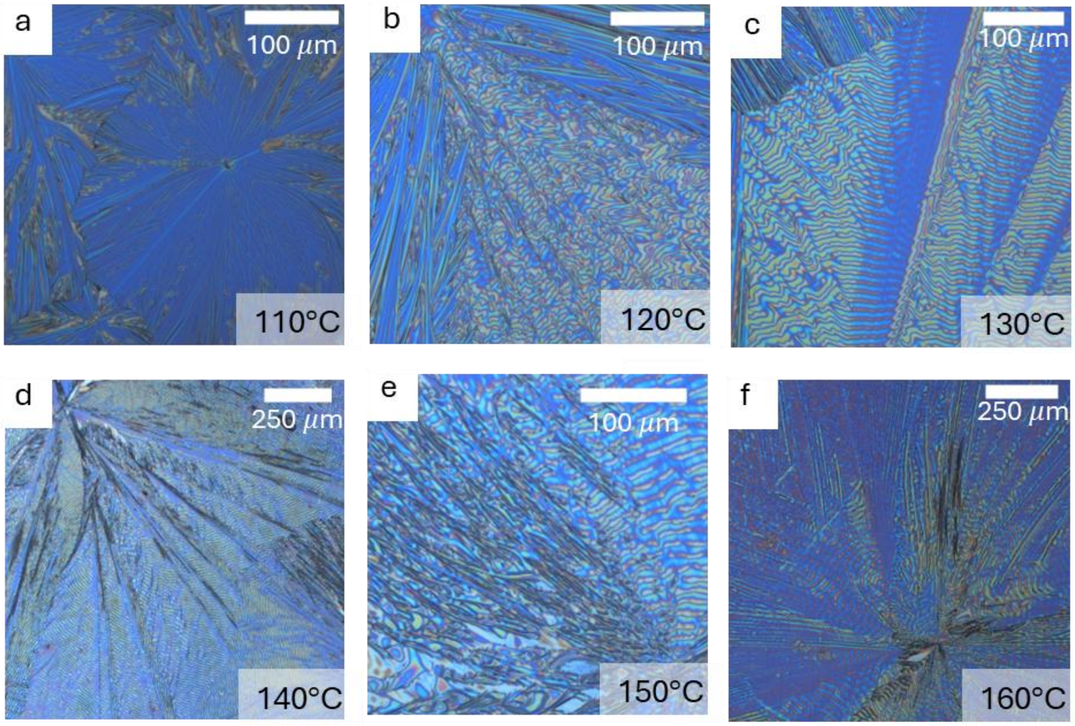

Figure S6. (a-f) Optical microscopy of (*R*-NEA)$_2$PbBr$_4$ films grown from DMF at temperatures from 110-160°C.

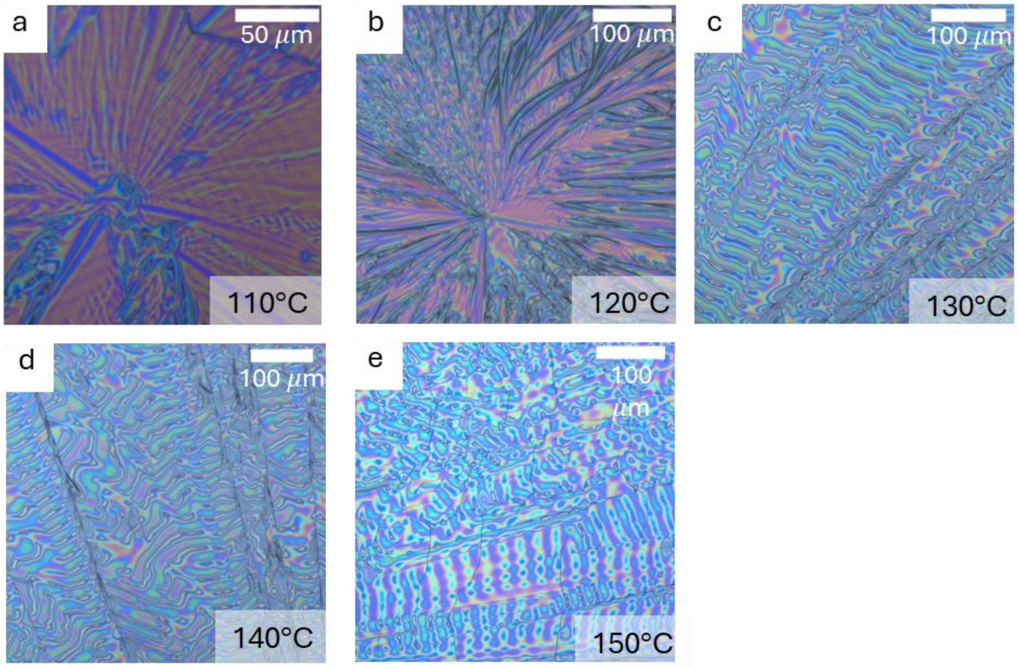

Figure S7. (a-e) Optical microscopy of (*R*-NEA)$_2$PbBr$_4$ films grown from 2-ME at temperatures from 110-150°C.

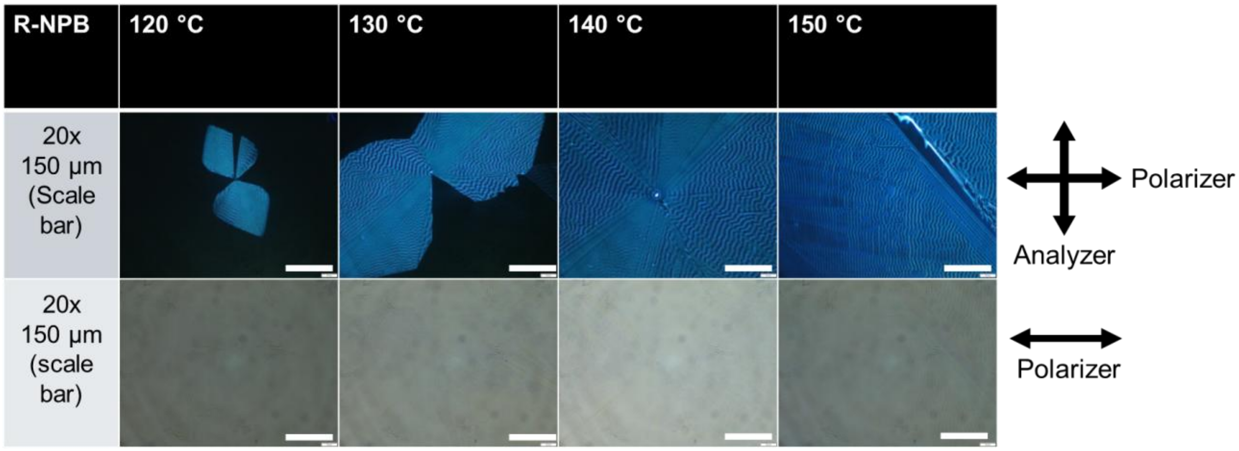

Figure S8. Cross polarized microscopy of (R-NEA)$_2$PbBr$_4$ grown at different temperatures and with different polarizer orientations.

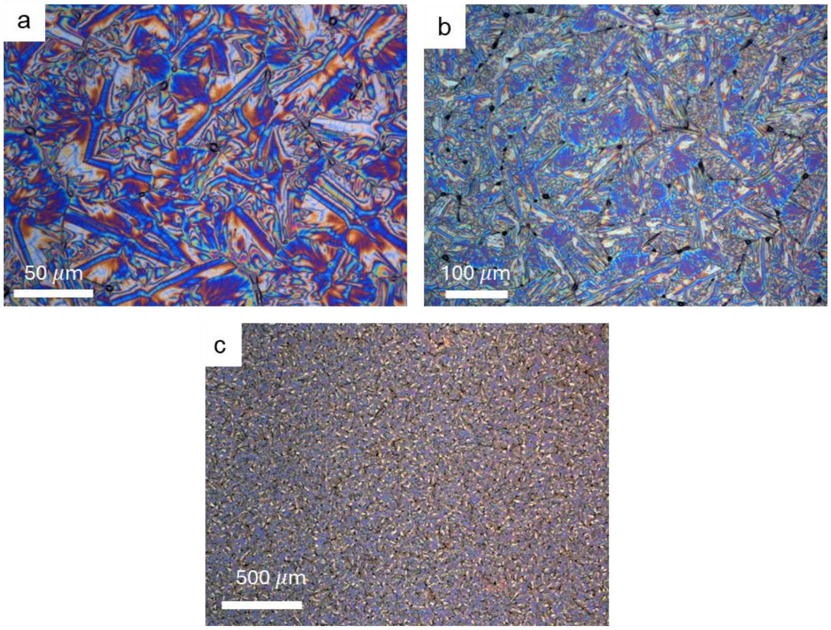

Figure S9. (a-c) Optical images of a spin coated film of racemic compound (*rac*-NEA)$_2$PbBr$_4$.

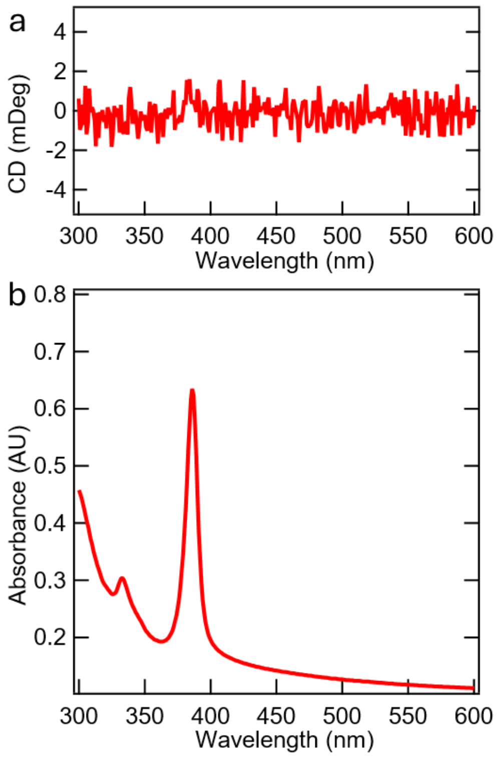

Figure S10. (a) Circular dichroism and (b) absorbance of (*rac*-NEA)$_2$PbBr$_4$.

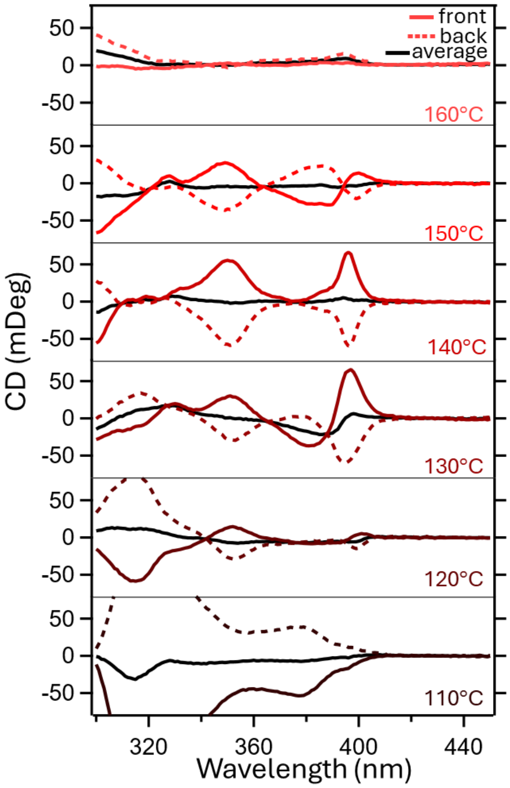

Figure S11. CD of (*S*-NEA)$_2$PbBr$_4$ processed from 2-ME at varying temperatures as noted on the plot.

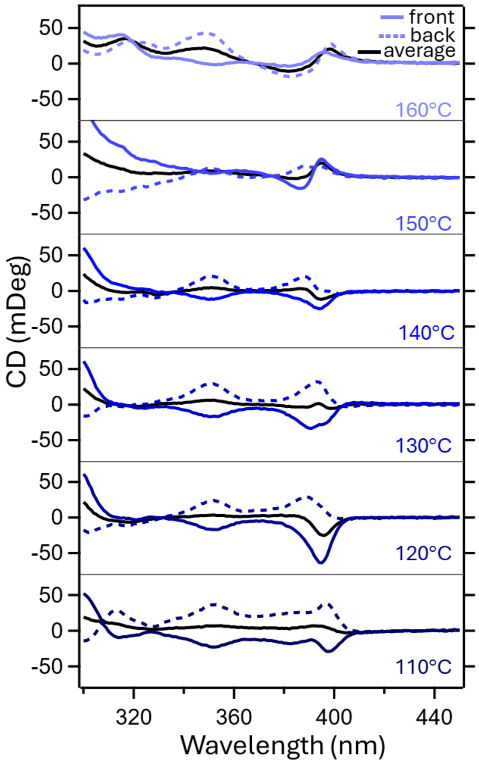

Figure S12. CD of (R-NEA)$_2$PbBr$_4$ processed from DMF at varying temperatures as noted on the plot.

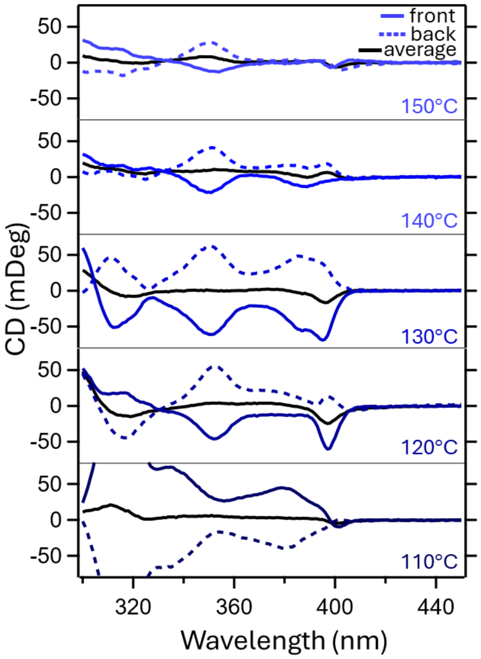

Figure S13. CD of (R-NEA)$_2$PbBr$_4$ processed from 2-ME at varying temperatures as noted on the plot.

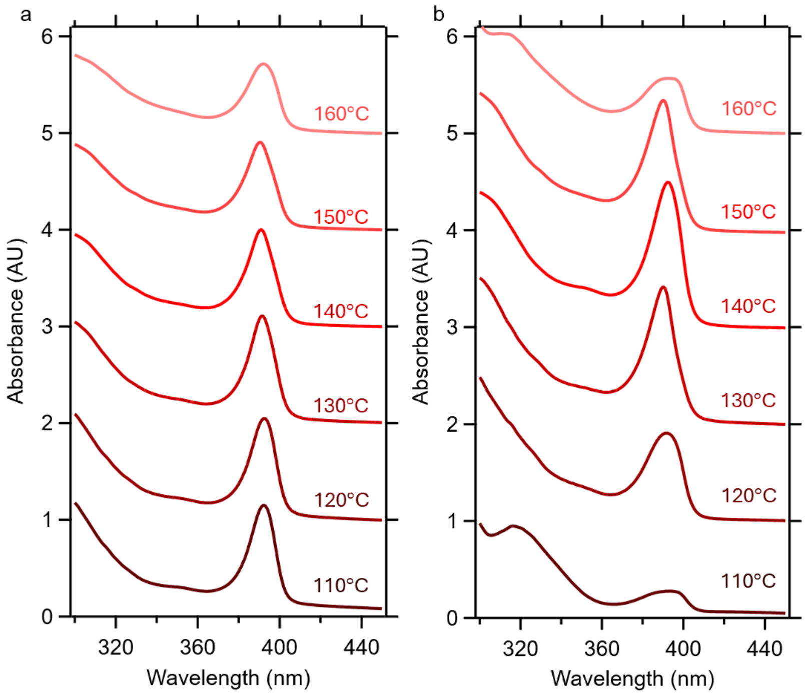

Figure S14. Absorbance of (S-NEA)$_2$PbBr$_4$ processed from (a) DMF and (b) 2-ME at varying temperatures as noted on the plot.

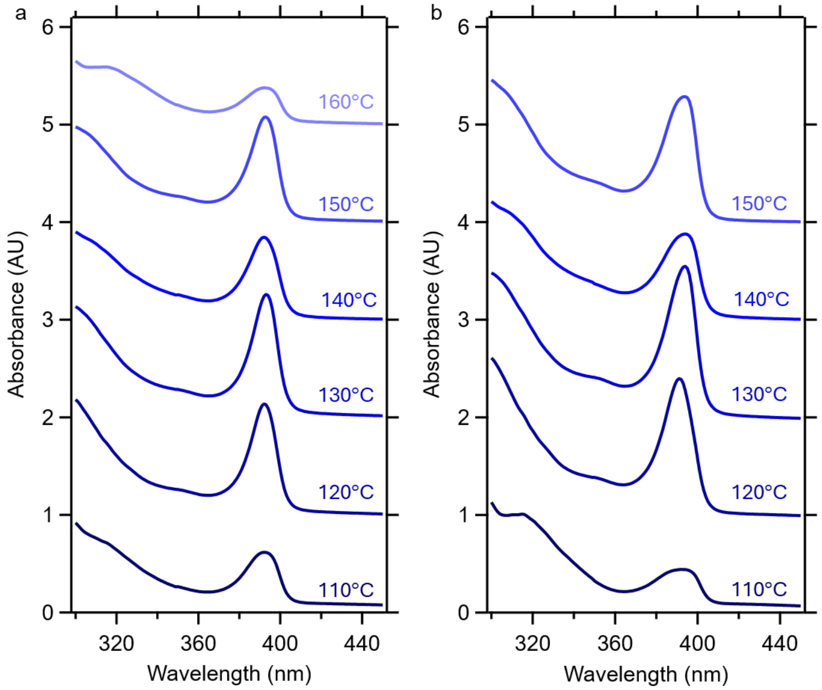

Figure S15. Absorbance of (R-NEA)$_2$PbBr$_4$ processed from (a) DMF and (b) 2-ME at varying temperatures as noted on the plot.

S2. Supplementary note on CD/apparent-CD model

S2-1. Introduction

Main text **Figure 4** and the supplementary figures above show CD spectra measured on $(S/R\text{-NEA})_2PbBr_4$ films grown with different annealing temperatures, all of which exhibit non-planar banded morphologies (**Figure 1**) while remaining crystallographically oriented, with the inorganic layers parallel to the substrate. As discussed in the main text, films processed using DMF exhibit a morphology consisting of symmetric ridge-valley structures (Figure 1 b,g) while films processed using 2-ME exhibit an asymmetric dune-like morphology (Figure 1c,h). Focusing on exciton absorption bands near 392nm displayed in **Figure 4**, measured on DMF-processed films, various distinct CD spectral signatures are observed.

The first obvious feature in **Figure 4** is that *all* of the spectra displayed are non-symmetric with respect to measurement on the front side versus the back (substrate) side of the samples. Comparison of measurements with light incident from the front side versus the back side is equivalent to reversal of the direction of light propagation with the sample held in fixed position. The lack of front/back symmetry in these spectra is not surprising since the film texture is not generally symmetric between the front side and the back (substrate) side, and refraction effects therefore come into play which are different between front-side and back-side measurement.

Figure 4 shows several examples of mono-signate CD spectra at the exciton resonance which is a characteristic of excitonic "apparent CD". This effect can occur when the exciton possesses non-degenerate levels whose electric transition dipoles are non-orthogonal and not parallel and can be described in terms of the "LDLB effect", wherein there is a misalignment between the linear dichroism and the linear birefringence in the sample.[1] However, another characteristic of apparent CD is that it is antisymmetric with respect to reversal of the direction of incident light or equivalently, flipping of the sample. Reviewing **Figure** 4, we find that some of these spectra show monosignate CD spectra when measured only from the front, or from the back, or when measured both from the front and the back. The average of the front/back spectra of some samples are monosignate, despite the common belief that apparent CD effects can be removed from thin film samples by averaging the CD spectra measured from the front and back side.

Furthermore, within **Figure 4** there are certain CD spectra which are particularly puzzling: For example, **Figure 4** shows CD spectra for films processed at 110C which are bisignate. At first glance these spectra appear to exhibit the Cotton effect as expected for intrinsic excitonic CD. However, excitonic intrinsic CD is symmetric with respect to direction reversal while these spectra are *antisymmetric* with respect to measurement on the front and the back sides (equivalent to reversal of the direction of light propagation relative to the sample normal). Another form of apparent CD can occur for double-layered structures with a relative twist about the optical axis.[1] However, this phenomenon is symmetric with respect to direction reversal while the measured spectra here are antisymmetric. This result is therefore anomalous.

In this note we discuss the mechanisms whereby the morphology depicted in **Figure 1** can give rise to such signatures in the exciton band of the CD spectra. We will start with a symmetry-based fine structure model for flat planar films. We will then show how various CD spectral shapes can be generated by the dune/ride morphology due to refraction effects and possible strain effects. We also consider the effect of interlayer twist which can give rise to bisignate spectra which are symmetric between measurement on the front and back.

S2-2. Crystal structure and exciton fine structure model for chiral *S/R* NPB

We begin by describing the crystal structure of chiral *S/R* NPB films and proceed to a symmetry-informed model for excitonic fine structure in these materials. This will be used to develop a model for excitonic CD in films that are planar with no strain or morphology. This will then be used as a vehicle to illustrate the effects of strain and morphology on the CD spectra.

S2-2-1. Crystal structure for chiral *S/R* NPB

Chiral *S/R* NPB crystallize in the non-symmorphic $P2_1$ space group, with a 2_1 screw axis in the plane of the inorganic layers. It has point group symmetry C_2. The crystal structures were previously reported.[2,3] The primitive vectors are given for *S*-NPB in **Table S1**. We adopt a coordinate system with cartesian coordinate *z* directed normal to the inorganic layers, with coordinates *x* and *y* spanning the plane of the inorganic layers and take the y direction as parallel to the 2_1 screw axis. The crystal structure is polar in the sense that the chiral distortion of the structure allows a polar distortion of the lead bromide octahedra, whose direction is parallel to the primitive vector $\boldsymbol{a_2}$ in S-NPB (**Table S1** panel **b**). The polar distortion can be shown to be oppositely directed, anti-parallel to primitive vector $\boldsymbol{a_2}$, in *R*-NPB.[4]

Table S1. Crystal structure and local formal dipole moments in *S*-NPB

Part (a) gives the primitive vectors of the experimentally determined structure of *S*-NPB, space group $P2_1$, as reported in Supplementary Table 35 of Ref.3. Vectors $\boldsymbol{a_1}$ and $\boldsymbol{a_2}$ lie in the plane of the inorganic layers with $\boldsymbol{a_2}$ parallel to the 2_1 screw axis, defining the y axis, with the x axis parallel to vector $\boldsymbol{a_1}$. Vector $\boldsymbol{a_3}$ is the layer stacking direction and points largely out of plane, with angle β between $\boldsymbol{a_1}$, $\boldsymbol{a_3}$ equal to 93.805 degrees. Part (b) shows the total local formal dipole associated with each Pb atom within a unit cell, and their resultant. The local dipole moment associated with each Pb site, set as origin, is calculated from the coordinates of the surrounding nearest six Br atoms using the general formula: $\sum q_{Br}\,(\boldsymbol{r_{Br}} - \boldsymbol{r_{Pb}})$ (here, q_{Br} is taken as $-1\ e$ where e is the elementary charge). Atomic positions in the table are given in Angstroms in cartesian coordinates. The corresponding average of the local polarization within each $PbBr_6$ octahedron is 8.9 μC/cm^2 directed along y, parallel to the 2_1 screw axis.

(a)

	x (Å)	y (Å)	z (Å)
$\boldsymbol{a_1}$	8.724	0	0
$\boldsymbol{a_2}$	0	7.930	0
$\boldsymbol{a_3}$	-1.290	0	19.398

(b)

	B-sites	Px (e-Å)	Py (e-Å)	Pz (e-Å)
local dipole	Pb1	0.124	0.200	-0.231
local dipole	Pb2	-0.124	0.200	0.231
**Sum (e-Å)**	Pb1+Pb2	**0.000**	**0.399**	**0.000**

S2-2-2. Exciton fine structure in chiral *S/R* NPB

It is known that the conduction and valence bands in chiral NPB are each 2-fold degenerate at the Brillouin zone center.[2] There are therefore four exciton fine structure levels. Based on the C_2 point symmetry we can write down the symmetries of these levels with the corresponding allowed components of the electric and magnetic dipole transition by inspection of the character table for point group C_2. These are given in **Table S2.** Symmetry requires that the four fine structure levels be characterized according to one of two irreducible representations ("irreps"), A and B. using the coordinate system defined in **Table S1** symmetry allows exciton levels with irrep A to possess non-vanishing electric and magnetic transition dipole moments in the y direction, while the B exciton levels have allowed electric and magnetic transition dipoles in the x and z directions.

Table S2. Electric & magnetic dipole transition matrix elements, point group C_2

The table gives the allowed electric and magnetic dipole transition matrix elements for excitons in a system of point symmetry C_2 with polar distortion along the y direction. Irreducible representation ("irrep") assignments are from Ref.[5]

Irrep	State	Electric transition dipole components P_i	Magnetic transition dipole components M_j
A	$\mathcal{Y}/\mathcal{D}$	P_y	M_y
B	$\mathcal{X}/\mathcal{Z}$	P_x , P_z	M_x , M_z

In a recent work, Ref. 4, a model for the exciton fine structure in chiral *S/R*-NPB was developed using effective mass theory, parameterized by density functional calculations. The basis of the model consists of splitting of the four exciton fine structure levels under the influence of the electron-hole exchange interaction, crystal field effects, and Rashba-like spin splitting calculated in Refs.2,3. By analysis of the spin textures computed via density functional theory for *S/R* NPB, and through analysis of the effect of the polar distortion discussed in Section S2-2-1, the fine structure model can be fully determined. Here we simply present the results of the analysis in **Table S3**. The key point is that the model reflected in **Table S3** reflects the symmetry of chiral NPB correctly. Inspection of the transition dipoles in the table and comparison with **Table S2** shows that the model is fully consistent with symmetry constraints. In particular we see that by virtue of the polar distortions in chiral *S/R*-NPB, magnetic dipole transition moments become allowed. In the C_2 point symmetry, these transition moments are not orthogonal to the electric dipole transition moments so that intrinsic CD becomes allowed. Moreover within each of the A and B exciton series, the rotary strengths for measurement normal to the inorganic layers, which can be shown to be proportional to $\mathrm{Im}\left(\tilde{p}_{n,x}\,\tilde{m}_{n,x} + \tilde{p}_{n,y}\,\tilde{m}_{n,y}\right)$ using the definitions in **Table S3**, are equal and opposite, so that a Cotton effect is expected. Finally, since the direction of the magnetic transition dipoles relative to the electric transition dipoles is reversed in *R*-NPB relative to *S*-NPB, tracking the direction of the polar distortion in the two materials, the intrinsic CD spectra for *S*-NPB and *R*-NPB have opposite polarity, as expected. This is illustrated below in **Figure S2- 1**.

In the next section we build upon this fine structure to develop a model for CD which can capture not only intrinsic CD but apparent CD effects as well.

Table S3. Comparison of fine structure in *S*- and *R*-NPB

The table compares the exciton fine structure level energies and electric and magnetic transition dipole moments in *S*-NPB, panel (**a**), and *R*-NPB, panel (**b**). Calculations include short- and long-range electron/hole exchange plus the effective exchange interaction due to Rashba-like spin-splitting for in-plane dispersion. The transition dipoles $\tilde{\boldsymbol{p}}_n$ and $\tilde{\boldsymbol{m}}_n$ are expressed in dimensionless form and are related to the dimensioned transition dipoles through $\boldsymbol{P}_n = i|P_K|\mathcal{K}\,\tilde{\boldsymbol{p}}_n$, and $\boldsymbol{M}_n = \hbar\mathcal{K}\,\tilde{\boldsymbol{m}}_n$ in terms of P_K, the Kane momentum matrix element and $\mathcal{K}$, an overlap factor, which is common to all fine structure levels. The relative magnitude, $\tilde{\mathcal{R}}_{CD,\perp}$, of the CD at normal incidence for each of the set of four fine structure states, n, given by $\tilde{\mathcal{R}}_{CD,\perp} = \mathrm{Im}(\tilde{p}_{n,x}\,\tilde{m}_{n,x} + \tilde{p}_{n,y}\,\tilde{m}_{n,y})$ is shown in the table. Panel (**a**) shows fine structure calculated for *S*-NPB using parameters given in Ref. 4. Panel (**b**) shows fine structure calculated for *R*-NPB, using the same parameters but reversing the sign of polar distortion relative to S-NPB. This sign reversal causes a sign change in the magnetic transition dipoles in *R*-NPB relative to *S*-NPB.

(a) *S*-NPB

State	Irrep	E (meV)	$\tilde{p}_x$	$\tilde{p}_y$	$\tilde{p}_z$	$\mathrm{Im}(\tilde{m}_x)$	$\mathrm{Im}(\tilde{m}_y)$	$\mathrm{Im}(\tilde{m}_z)$	$\tilde{\mathcal{R}}_{CD,\perp}$
$\mathcal{Z}$	B	51.74	0.047	0	0.476	-0.315	0	0.017	-0.015
$\mathcal{Y}$	A	22.61	0	0.979	0	0	-0.031	0	-0.030
$\mathcal{X}$	B	13.12	0.797	0	-0.028	0.019	0	0.283	0.015
$\mathcal{D}$	A	7.64	0	-0.221	0	0	-0.137	0	0.030

(b) *R*-NPB

State	irrep	E (meV)	$\tilde{p}_x$	$\tilde{p}_y$	$\tilde{p}_z$	$\mathrm{Im}(\tilde{m}_x)$	$\mathrm{Im}(\tilde{m}_y)$	$\mathrm{Im}(\tilde{m}_z)$	$\tilde{\mathcal{R}}_{CD,\perp}$
$\mathcal{Z}$	B	51.74	0.047	0	0.476	0.315	0	-0.017	0.015
$\mathcal{Y}$	A	22.61	0	0.979	0	0	0.031	0	0.030
$\mathcal{X}$	B	13.12	0.797	0	-0.028	-0.019	0	-0.283	-0.015
$\mathcal{D}$	A	7.64	0	-0.221	0	0	0.137	0	-0.030

S2-3. Model for intrinsic circular dichroism, planar films

With a symmetry-constrained model for the exciton fine structure in chiral NPB in hand, we can develop a model for the excitonic CD. This is accomplished by using the exciton fine structure to build an expression for the complex dielectric response of the material, with corrections for magnetic dipole transitions. The relative dielectric tensor response of the exciton for light propagating along direction $\hat{k}$, including both electric and magnetic transition dipole contributions,[4] can be written as,

$$\boldsymbol{\epsilon}(E) = \epsilon_\infty \left\{ \mathbf{1} + \Delta_{LT} \sum_n \mathcal{L}(E, E_n) \left(\tilde{\boldsymbol{p}}_n^{\,*} \otimes \tilde{\boldsymbol{p}}_n + h_m\, \tilde{\boldsymbol{p}}_n^{\,*} \otimes (\tilde{\boldsymbol{m}}_n \times \hat{k}) + h_m (\tilde{\boldsymbol{m}}_n \times \hat{k})^* \otimes \tilde{\boldsymbol{p}}_n \right) \right\}. \tag{S1}$$

Here, ϵ_∞ is the background non-resonant contribution, $\mathbf{1}$ is the 3x3 identity matrix, Δ_{LT} is the exciton longitudinal-transverse splitting parameter which determines the strength of the light-matter interaction. The sum is taken over the set of four fine structure levels, n, with energy E_n and dimensionless electric and magnetic transition dipoles $\tilde{\boldsymbol{p}}_n$ and $\tilde{\boldsymbol{m}}_n$, respectively. Each term in the sum has a complex Lorentzian response, $\mathcal{L}(E, E_n)$, centered at E_n; the

symbol ⊗ denotes the Kronecker product, and the term h_m represents the ratio of the strength of the magnetic to the electric dipole light interactions. It is given by,

$$h_m \equiv \frac{\hbar k}{2|P_K|}\ , \tag{S2}$$

where k is the norm of the wave vector of the light wave.

To compute the optical properties we insert the tensor dielectric function into the wave equation and solve for the normal modes for transverse light propagation, To analyze transverse wave propagation along the z direction normal to the layers, we write the wave equation in terms of the transverse components of the electric field resulting in a reduced dielectric tensor[6,7] from which we determine the normal modes, which are, in general, elliptically polarized. We note that the dielectric tensor is complex, and is neither Hermitian nor symmetric, making diagonalization by spatial rotation operations impossible as the real and imaginary parts of the tensor have different principle axes.[8] Moreover the normal modes are not orthonormal in the usual sense. We are nevertheless able to perform a normal mode decomposition for incident light of arbitrary polarization by exploiting the fact that the propagating eigenmodes are *biorthogonal* with the set of adjoint eigenmodes, which are eigenmodes of the Hermitian adjoint of the wave equation.[4,9,10] Then the decadic absorbance $\mathbb{A}$ for incident light of a given polarization $\hat{\boldsymbol{e}}_{inc}$ is computed for a sample of thickness L as,

$$\mathbb{A}(\hat{\boldsymbol{e}}_{inc}) = -Log_{10}\left[\left|\frac{\boldsymbol{E}_{out}(L)}{\boldsymbol{E}_{inc}}\right|^2\right]. \tag{S2}$$

The CD is then computed as the difference in decadic absorbance of incident light of left-and right- circular polarization. We note that utilization of the biorthogonality properties of the propagating normal modes allows effects such as "apparent CD" to be modelled directly from the wave equation rather than within the phenomenological Muller matrix approach, as implemented in, e.g., Ref.1, since the interplay of dispersion and loss effects are fully captured by the wave equation approach. The quantitative accuracy of the phenomenological approach developed in Ref. 1 is limited due to approximations made in estimating the linear dichroism and linear birefringence from the complex dielectric tensor. Specifically, it was assumed in Ref. 1 that components of the complex refractive index tensor can be computed as the square root of the components of the dielectric matrix. This is not an admissible procedure in general since the components of the square root of a given matrix are not equal to the square root of the matrix components unless the matrix is diagonal, as can be directly verified.

Computed CD spectra for planar 120 nm thick films of *S/R* NPB are shown in **Figure S2-1.** As expected the CD spectra exhibit a bisignate Cotton effect, are symmetric with respect to reversal of the direction of light propagation, and have opposite polarity for *S*-NPB and *R*-NPB. The polarity of the spectra for *S*-NPB agrees with that determined for Im $\boldsymbol{\epsilon}$ by ab initio calculations and analysis of reflection from single crystal *S*-NPB in Ref. 11.

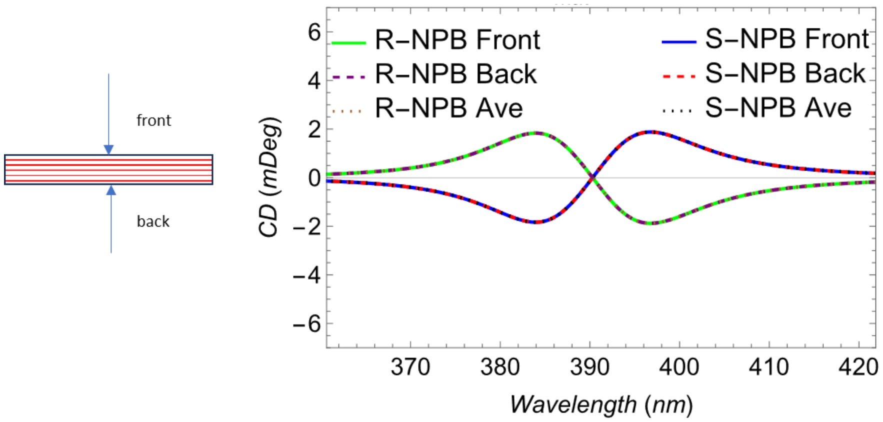

Figure S2- 1. CD spectra for planar films of chiral *S/R* NPB
Spectra are computed using the fine structure parameters of **Table S3**. Other parameters in the calculation: film thickness L=120nm; Transition linewidth (full width at half maximum) LW=150 meV; light-matter interaction strength Δ_{LT}= 53 meV; background high frequency relative permittivity ϵ_∞ =3.65; ratio of magnetic to electric coupling strength h_m= 0.0033. See Ref. 4 for details.

S2-4. Models for circular dichroism in non-planar films

We now address the question of the origin of the various CD spectral signatures measured on textured films reported in **Figure 4**. We will first consider the effects of interlayer twist which can give rise to bisignate spectra which are symmetric between measurement on the front and back. We will then show how various CD spectral shapes can be generated by the dune/ridge morphology due to refraction and possible strain effects.

S2-4-1. Interlayer twist

Figure S2- 2 depicts model calculations of the effect of interlayer twist within a thin film of *S*-NPB. Here it is assumed that the thin film is comprised of two stacked grains, both crystallographically aligned with their inorganic layers parallel to the substrate and normal to the optical axis with equal thickness. The top grain has crystallographic $\boldsymbol{a_1}$-direction at a twist angle γ relative to that of the bottom grain as shown in the figure. This sort of grain twist gives rise to a bisignate CD spectrum which is symmetric with respect to illumination from the top of bottom side of the stack. The spectral shape is similar to that of intrinsic CD (**Figure S2- 1**) but has much larger magnitude and has a polarity which is dependent on the sense of the relative twist angle. A small twist may explain the CD spectra in Fig S10, processed at 160C, which exhibit a symmetric, bisignate excitonic CD response whose polarity is reversed from that of the intrinsic CD of planar films.

Interlayer twist can also give rise to antisymmetric bisignate response as shown in **Figure S2- 3**. There, a three-layer structure is modelled with the in-plane crystallographic orientation of the middle layer rotated by angle γ relative to the top and bottom layers. However, the peak CD signal in this case is 100 times smaller than the double layer case at the maximal interlayer twist of 45 degrees. This scenario is not likely to explain the antisymmetric bisignate response of the 110C sample shown in **Figure 4** of the main text.

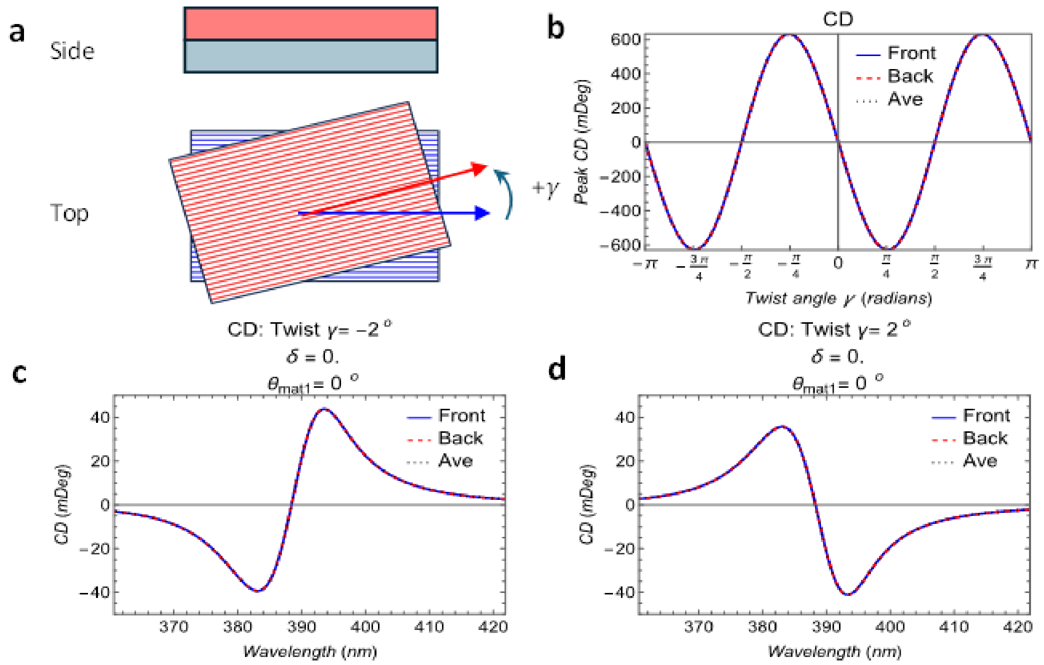

Figure S2- 2. Exciton CD response of two stacked grains.
The top grains' in-plane crystallographic directions $\boldsymbol{a_1}$ is rotated relative to that of the bottom grain by twist angle γ (panel **a**). Spectra exhibit a symmetric bisignate CD response whose polarity depends on the angle of relative twist between the two layers (panels **c, d**). The CD response at the red-side lobe of the bisignate CD spectrum is plotted in panel **b**. The peak magnitude is maximum for twist angles of ± 45 and ± 135 degrees. Material parameters are the same as in **Figure S2- 1**, for *S*-NPB.

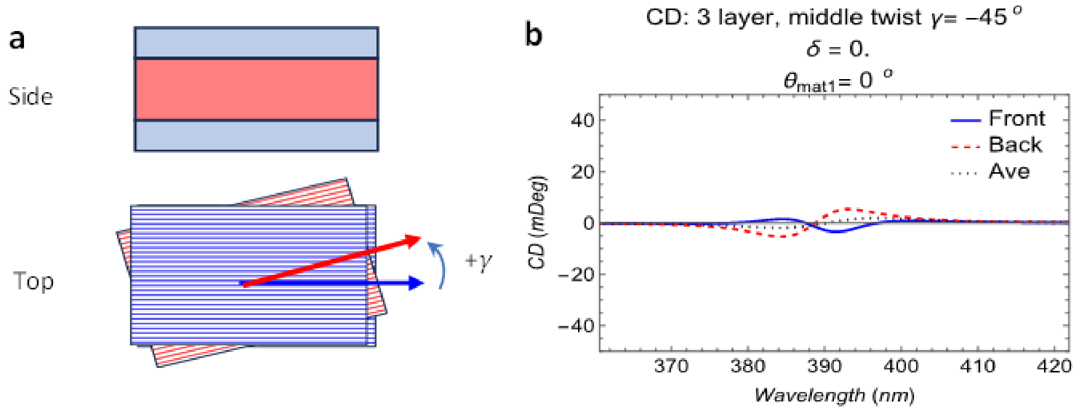

Figure S2- 3. Exciton CD response of three stacked grains.
In the simulation, the middle layer has its in-plane crystallographic orientation is twisted by angle γ relative to the top and bottom layers (panel **a**). Spectra exhibit a bisignate CD response antisymmetric with respect to direction of illumination, and whose polarity depends on the angle of relative twist between the layers (panel **b**). In the simulation the thickness of the top/middle/bottom layers are in the ratio 1:2:1. The spectrum is plotted for -45 degree twist: The magnitude in this case is 2 orders of magnitude smaller than for the two stacked layers shown in **Figure S2-2**. Calculations are performed using the parameters for *S*-NPB in **Table S3** and **Figure S2- 1**.

S2-4-2. Refraction effects.

As discussed in the main text, films processed using DMF exhibit a morphology consisting of symmetric ridge-valley structures (Figure 1 **b, g**) while films processed using 2-ME exhibit an asymmetric dune-like morphology (Figure 1 **c, h**). Both these cases will affect the CD response because the textured morphology caused light to refract into the sample so that it propagates at an angle θ_{mat} away from the axis normal to the inorganic layers. This is depicted in **Figure S2-4** for dune and ridge structures. Because in general the top and back (substrate) sides of the films can be expected to have different morphology, we expect the CD spectra to be non-symmetrical when measured from the front and the back, which is generally the case in spectra displayed in **Figure 4**, for example.

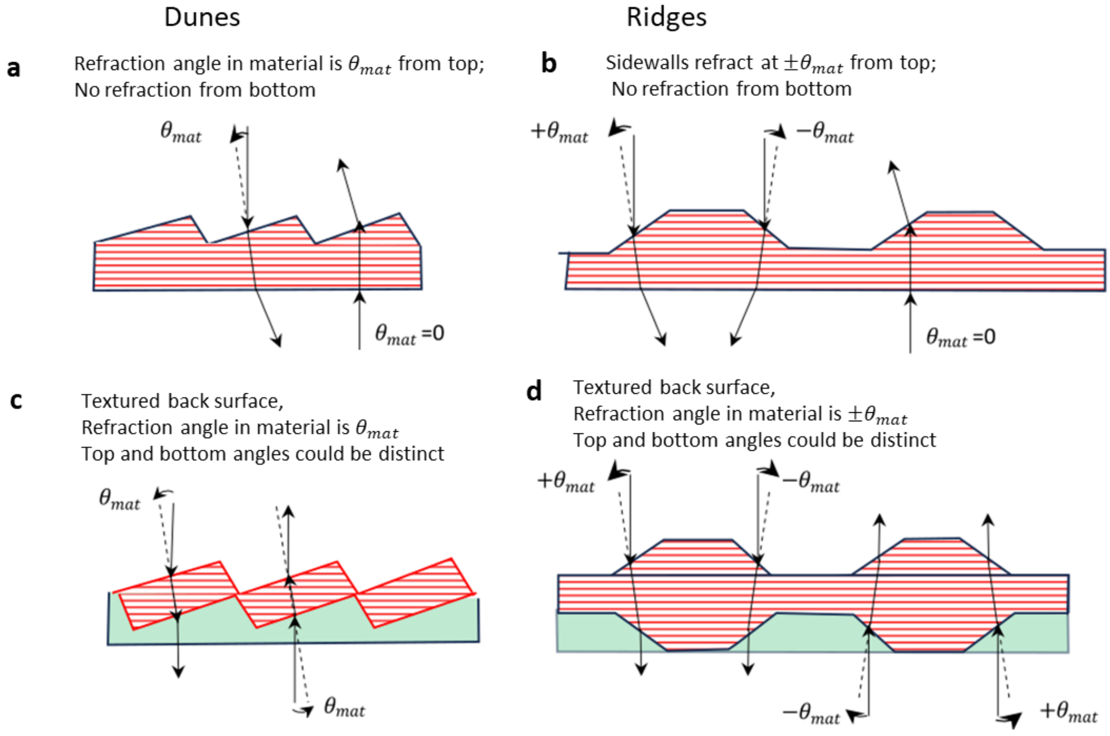

Figure S2- 4. Model depiction of refraction in films with dune- and ridge-like morphology.
Panels **a, b** depict refraction effects in dune and ridge structures with a flat back (substrate) side. Light incident on angled sidewall features refracts at some angle θ_{mat} away from the z-axis normal to the inorganic layers. Panels **c, d** depict possible morphologies where dune- and ridge-like texture exists on both the front and back sides. The refraction angles for backside illumination are drawn symmetric with the front-side illumination. Although front/back symmetry is unlikely in actual samples, it is a useful construction for modelling to understand symmetry properties of the CD response under sample flipping or light direction reversal. Horizontal red lines depict crystallized grains with inorganic layers parallel to the substrate while solid green depicts amorphous material assumed to be of low density and lower refractive index that crystallized regions.

Now we consider the question of how refraction might cause antisymmetric “apparent-CD” effects.

In Ref. 1 it was shown that excitonic apparent CD can occur when the exciton possesses non-degenerate levels whose electric transition dipoles are non-orthogonal and not parallel. Reference to **Table S3** shows that indeed, the electric dipole transition vectors of the two non-degenerate fine structure levels corresponding to irreducible representation B are neither orthogonal nor parallel. However, for light propagating normal to the plane of the inorganic layers, the projection of the dipole transition vectors for these two states into the plane of the

inorganic layers are orthogonal, so that excitonic apparent CD effects do not occur. Neither are apparent CD effects expected at normal incidence for the two non-degenerate fine structure levels corresponding to irreducible representation A whose electric dipoles are co-linear.

That situation changes if the light is propagating at an angle θ_{mat} from the z-axis, however. In that case, the electric field vector of the light can couple not just to the x- and y- components of the electric dipole transition vectors, but to the z-component as well. We can visualize the potential for excitonic apparent-CD easily by expressing the electric and magnetic transition dipoles for each fine structure state in a rotated coordinate system, x', y', z', where the z' axis is parallel to the wave vector of the refracted light, and the x', y' coordinates span the plane normal to the light wave vector. Then the assessment of whether apparent CD should occur is made by examination of the angles between the projections of the electric transition dipoles in the x', y' plane. To perform this transformation we visualize a ridge or dune structure whose ridge-top or dune-crest is aligned to x' at an azimuth angle ϕ relative to the crystallographic direction $\boldsymbol{a_1}$ (**Figure S2-5**). Then the angle of propagation is θ_{mat} rotated about the x' axis.

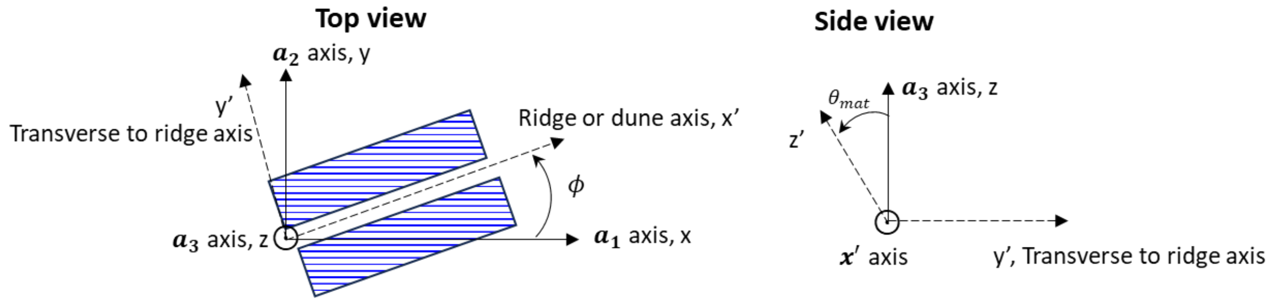

Figure S2- 5. Model depiction of coordinates to describe refraction in films with dune- and ridge-like morphology.
Dune or ridge crests are aligned to coordinate x' which is rotated by angle ϕ from the crystallographic direction $\boldsymbol{a_1}$. Light incident on the angled sidewall features refracts at some angle θ_{mat} away from the z-axis normal to the inorganic layers.

The results of this procedure are given in **Table S4**. There we see that rotation by an angle θ_{mat} about any azimuth other than 90 degrees gives rise to non-orthogonal, non-parallel electric transition dipole vectors in the x',y' plane. Since the levels are non-degenerate, these rotations are prescriptive for observation of excitonic apparent CD as described by Salij *et al.* in Ref. 1. Notably, for the 90 degree azimuths, the projected electric dipole vectors in the x' y' plane are all at either 0 or 90 degrees from one another. Consequently, for that case excitonic apparent CD effects are not expected. Nevertheless, there is an enhancement of the rotary strength due to a distinct effect: Three-dimensional (3D) extrinsic CD.[12] The CD in this case is monosignate, symmetric with respect to reversal of the direction of light, but antisymmetric with respect to rotation angle θ_{mat}. This phenomenon occurs even in orthorhombic systems but vanishes if the magnetic dipole transition moments are set to zero. This will be discussed further below.

Table S4. Transition dipoles for *S*-NPB in rotated coordinates

The table shows the exciton fine structure level energies and electric and magnetic transition dipole moments in *S*-NPB, when expressed in rotated coordinates x',y',z' aligned to a light wave refracted by angle θ_{mat} from the z-axis around an axis x' rotated by azimuth angle angle ϕ relative to the crystallographic direction $\boldsymbol{a_1}$. The transition dipoles $\tilde{\boldsymbol{p}}_n$ and $\tilde{\boldsymbol{m}}_n$ are expressed in dimensionless form and are related to the dimensioned transition dipoles through $\boldsymbol{P}_n = i|P_K|\mathcal{K}\,\tilde{\boldsymbol{p}}_n$, and $\boldsymbol{M}_n = \hbar\mathcal{K}\,\tilde{\boldsymbol{m}}_n$ in terms of P_K, the Kane momentum matrix element and $\mathcal{K}$, an overlap factor, which is common to all fine structure levels. The relative magnitude, $\tilde{\mathcal{R}}_{CD,}(\theta_{mat}, \phi)$ of the CD for each of the set of four fine structure states, n, given by $\tilde{\mathcal{R}}_{CD,}(\theta_{mat}, \phi) = \mathrm{Im}\big(\,\tilde{p}_{n,x'}\,\tilde{m}_{n,x'} + \tilde{p}_{n,y'}\,\tilde{m}_{n,y'}\big)$ is shown in the table. The angles in degrees (deg) between the projections into the x', y' plane of the electric dipole transition vectors are given for all pairs of states.

(a) No refraction *Angles in x,y plane (deg)*

State	E (meV)	$\tilde{p}_{x'}$	$\tilde{p}_{y'}$	$\tilde{p}_{z'}$	$\tilde{m}_{x'}$	$\tilde{m}_{y'}$	$\tilde{m}_z{}'$	$\tilde{\mathcal{R}}_{CD,}$		$\mathcal{Z}$	$\mathcal{Y}$	$\mathcal{X}$	$\mathcal{D}$
$\mathcal{Z}$	51.74	0.047	0	0.476	-0.32 i	0 i	0.017 i	-14.8	$\mathcal{Z}$	0	90.0	180.0	90.0
$\mathcal{Y}$	22.61	0	-0.98	0	0 i	0.03 i	0 i	-30.4	$\mathcal{Y}$	90.0	0	90.0	0.0
$\mathcal{X}$	13.12	-0.8	0	0.028	-0.02 i	0 i	-0.28 i	14.8	$\mathcal{X}$	180.0	90.0	0	90.0
$\mathcal{D}$	7.64	0	-0.22	0	0.	-0.14i	0.	30.4	$\mathcal{D}$	90.0	0.0	90.0	0.0

(b) rotation by 20 degrees about x (0 degree azimuth) *Angles in x,y plane (deg)*

State	E (meV)	$\tilde{p}_{x'}$	$\tilde{p}_{y'}$	$\tilde{p}_{z'}$	$\tilde{m}_{x'}$	$\tilde{m}_{y'}$	$\tilde{m}_z{}'$	$\tilde{\mathcal{R}}_{CD}$		$\mathcal{Z}$	$\mathcal{Y}$	$\mathcal{X}$	$\mathcal{D}$
$\mathcal{Z}$	51.74	0.047	0.163	0.447	-0.32 i	0.006 i	0.02 i	-13.9	$\mathcal{Z}$	0	163.9	105.4	163.9
$\mathcal{Y}$	22.61	0	-0.92	0.335	0 i	0.03 i	-0.01 i	-26.8	$\mathcal{Y}$	163.9	0	90.7	0.0
$\mathcal{X}$	13.12	-0.8	0.01	0.026	-0.02 i	-0.01i	-0.27 i	13.9	$\mathcal{X}$	105.4	90.7	0	90.7
$\mathcal{D}$	7.64	0	-0.21	0.076	0.	-0.13 i	0.05 i	26.8	$\mathcal{D}$	163.9	0.0	90.7	0.0

(c) rotation by 20 degrees about 45 degree azimuth *Angles in x,y plane (deg)*

State	E (meV)	$\tilde{p}_{x'}$	$\tilde{p}_{y'}$	$\tilde{p}_{z'}$	$\tilde{m}_{x'}$	$\tilde{m}_{y'}$	$\tilde{m}_z{}'$	$\tilde{\mathcal{R}}_{CD}$		$\mathcal{Z}$	$\mathcal{Y}$	$\mathcal{X}$	$\mathcal{D}$
$\mathcal{Z}$	51.74	0.033	0.132	0.459	-0.22 i	0.22i	-0.06 i	20.9	$\mathcal{Z}$	0	147.4	60.5	147.4
$\mathcal{Y}$	22.61	-0.69	-0.65	0.237	0.02 i	0.02 i	-0.01 i	-28.6	$\mathcal{Y}$	147.4	0	87.0	0.0
$\mathcal{X}$	13.12	-0.56	0.539	-0.17	-0.01 i	-0.09 i	-0.27 i	-38.2	$\mathcal{X}$	60.5	87.0	0	87.0
$\mathcal{D}$	7.64	-0.16	-0.15	0.054	- 0.1 i	- 0.1 i	0.033 i	28.6	$\mathcal{D}$	147.4	0.0	87.0	0.0

(d) rotation by 20 degrees about x (90 degree azimuth) *Angles in x,y plane (deg)*

State	E (meV)	$\tilde{p}_{x'}$	$\tilde{p}_{y'}$	$\tilde{p}_{z'}$	$\tilde{m}_{x'}$	$\tilde{m}_{y'}$	$\tilde{m}_z{}'$	$\tilde{\mathcal{R}}_{CD}$		$\mathcal{Z}$	$\mathcal{Y}$	$\mathcal{X}$	$\mathcal{D}$
$\mathcal{Z}$	51.74	-0.12	0	0.46	-0.30 i	0 i	-0.09 i	35.8	$\mathcal{Z}$	0	90.0	0.0	90.0
$\mathcal{Y}$	22.61	0	-0.98	0	0 i	0.03 i	0 i	-30.4	$\mathcal{Y}$	90.0	0	90.0	0.0
$\mathcal{X}$	13.12	-0.76	0	-0.25	0.08 i	0 i	-0.27 i	-60.3	$\mathcal{X}$	0.0	90.0	0	90.0
$\mathcal{D}$	7.64	0	-0.22	0	0.	-0.14 i	0.	30.4	$\mathcal{D}$	90.0	0.0	90.0	0.0

S2-4-2-1. Refraction effects: Dune morphology.

To illustrate the effect the effect of refraction, we show calculated results for the dune morphology in **Figure S2-6**. For these calculations, in order to illustrate the antisymmetric response of the apparent CD effect with respect to reversal of incident light direction, we simulate a dune structure which is symmetric from top to bottom (geometry depicted in **Figure S2-6** panel **a**) although this is probably not likely in real samples**.** Calculations in **Figure S2-6** show that the spectral signature is generally monosignate and antisymmetric with respect to sample flipping as expected (panel **c**). The maximal CD response occurs when the dune azimuth is rotated ± 45 or ± 135 degrees from crystallographic direction $\boldsymbol{a_1}$ (panel **b**), with apparent CD contributions vanishing at azimuths of ± 90 degrees, as expected from **Table S4**, panel **d**. The response is not symmetric for positive and negative values of the dune azimuth angle ϕ and indeed reverses sign with sign reversal of refraction angle θ_{mat} at $\phi = 0$. This is illustrated in **Figure S2- 7** panels **a, b**. Thus, for example, in a symmetric ridge geometry, apparent CD response would cancel out for a ridge aligned with azimuth angle $\phi = 0$.

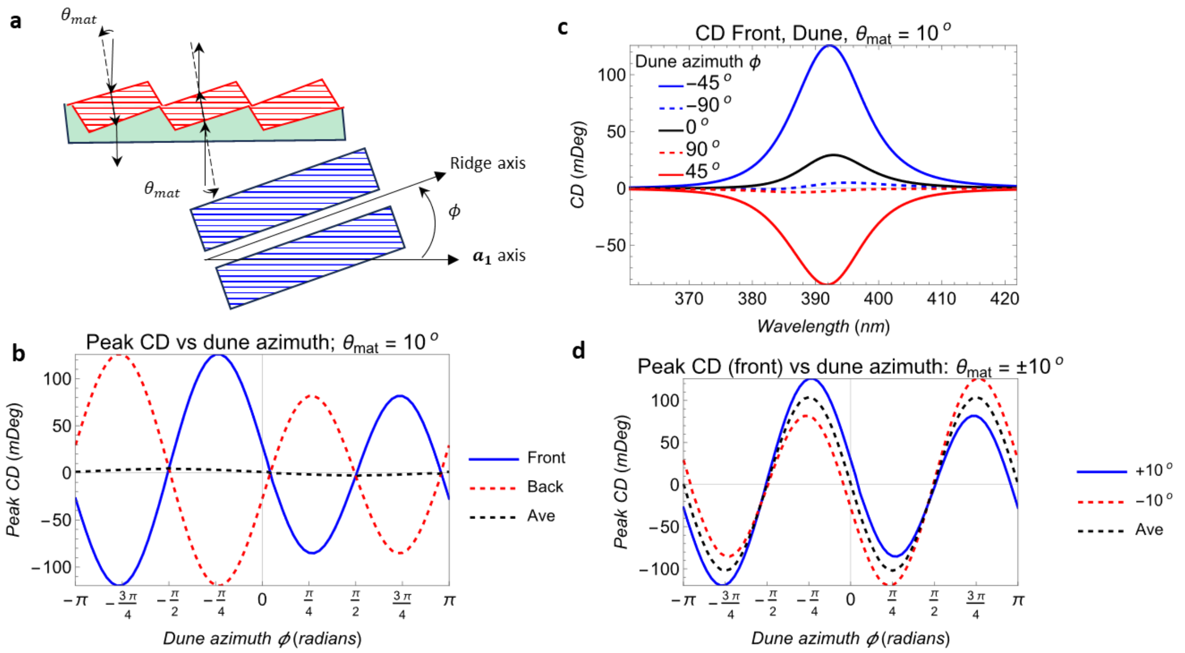

Figure S2- 6. Apparent CD response for dunes.
Panel **a**, model of geometry considered. Dune structure is taken schematically to be symmetric from top to bottom to illustrate directional antisymmetry. Horizontal red lines depict inorganic layers parallel to the substrate while solid green depicts low index amorphous material. Panel **b** shows peak CD versus azimuth angle ϕ for fixed θ_{mat}=+10 degrees. Panel **c** shows CD spectra from front side illumination at various azimuth angles and fixed θ_{mat}=+10 degrees. Panel **d** shows peak CD from frontside illumination versus azimuth angle ϕ for θ_{mat}= +10 degrees and $\theta_{mat} = -10$ degrees. Calculations are performed for *S*-NPB using material parameters in **Figure S2-1**.

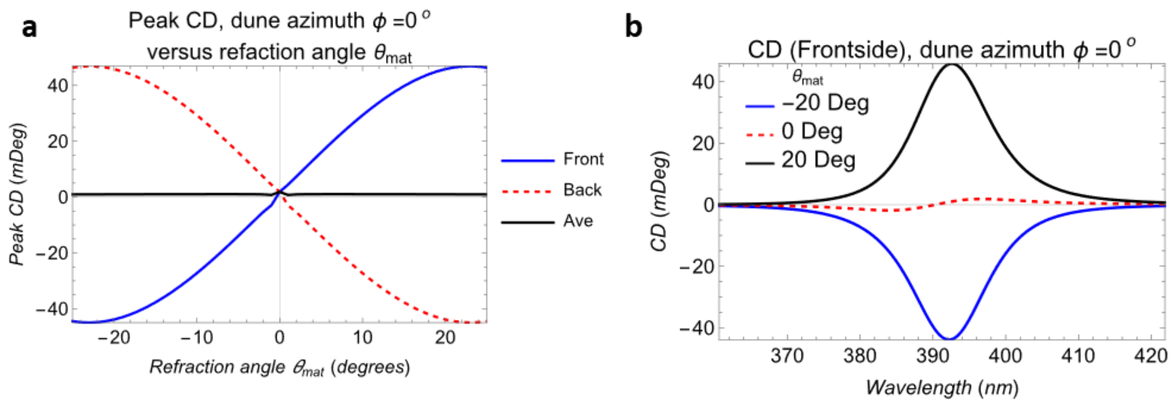

Figure S2- 7. Apparent CD response for dunes at dune azimuth ϕ=0 degree.
Structure and material parameters are the same as in **Figure S2- 6**. Panel **a** shows peak CD versus refraction angle θ_{mat}. Panel **b** shows CD spectra from front side illumination for θ_{mat}= +10 degrees and $\theta_{mat} = -10$ degrees.

3D extrinsic CD effect.

On the orthogonal dune axis, azimuth angle $\phi = 90$ degrees, which is aligned to y, the behavior, shown in **Figure S2- 8** panels **a, b**, is quite different that in **Figure S2-7** at 0 degree azimuth: For the 90 degree azimuth the CD is symmetric for front and back illumination (panel **a**) yet is monosignate for non-zero refraction angle θ_{mat} (panel **b**). This behavior is distinct from and not due to "apparent CD" as described in Ref. 1 since **Table S4** panel **d** shows that the projected dipoles in the plane of the electric field of the propagating light wave are mutually orthogonal. Rather, it is due to a distinct phenomenon, three-dimensional (3D) extrinsic CD.[12] If the magnetic dipole (MD) transition moments are turned off, the CD response vanishes. Conversely if the magnetic transition dipoles are non-zero, this effect occurs in a system of orthorhombic C_{2v} symmetry with polar axis along y. This is demonstrated in **Figure S2- 9** panels **a**,**b**. The enhanced CD response in that case is due to the fact that an incident light-wave at non-zero θ_{mat} in the plane of incidence containing the 90 degree azimuth (aligned to y) breaks the vertical mirror symmetry: the system comprising the sample plus the incident light is therefore chiral even though the sample is not. If the MD terms are set to zero, the sample has symmetry D_{2h}, and the CD response vanishes, as shown in **Figure S2- 9** panels **c**,**d.**

While interesting as a distinct phenomenon the 3D extrinsic CD effect is small in magnitude so we next return to discussion of apparent CD on the azimuths for which the apparent CD effect is maximized.

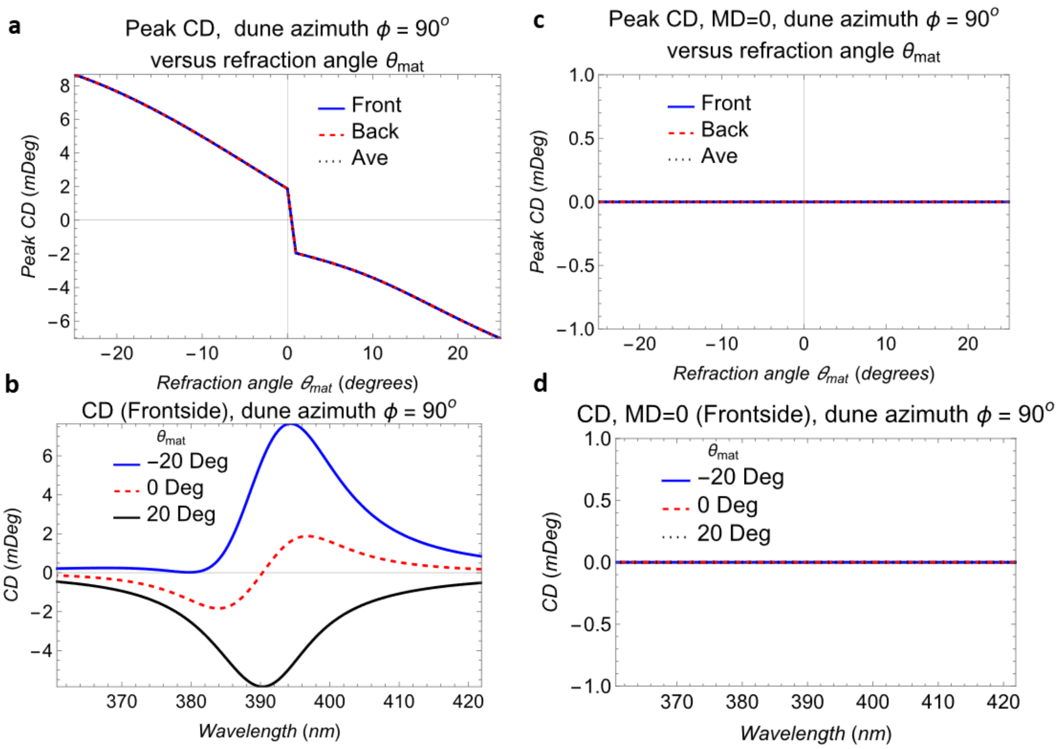

Figure S2- 8. 3D extrinsic CD response for dunes at dune azimuth ϕ=90 degree.
Structure and material parameters are the same as in **Figure S2- 6**. Panel **a** shows peak CD versus refraction angle θ_{mat}. Panel **b** shows CD spectra with front side illumination for $\theta_{mat}= \pm 20$ degrees and $\theta_{mat} = 0$degrees. With non-zero refraction angles, the CD is monosignate, symmetric with respect to sample flipping, but antisymmetric with respect to the sign of the refraction angle. Panels **c**, **d** repeat the calculations of panel **a, b** but with the magnetic dipole (MD) transition moments set to zero. For these cases, there is no CD response.

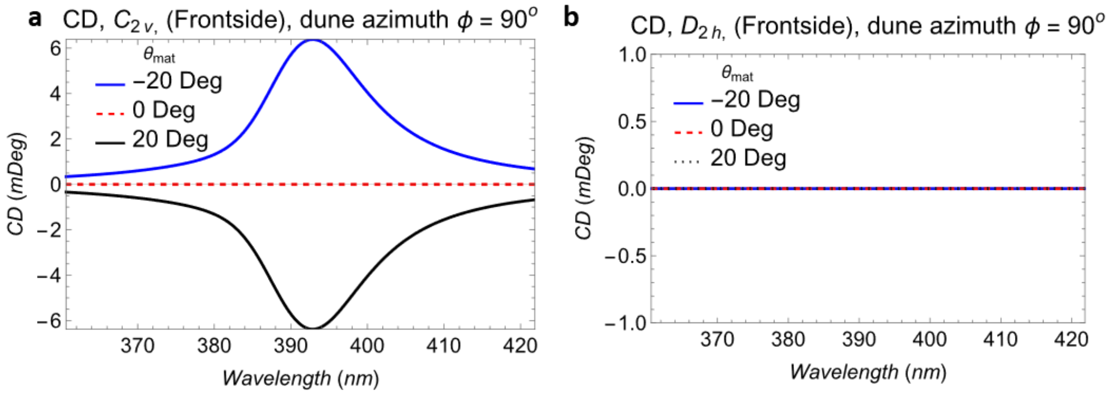

Figure S2- 9. 3D extrinsic CD response for dunes at dune azimuth ϕ=90 degree, symmetry C_{2v} and D_{2h}.
Geometry is the same as in **Figure S2- 6** panel **a** but in this case the material has orthorhombic symmetry. Panel **a** shows CD spectra with front side illumination for $\theta_{mat}= \pm 20$ and 0 degrees for sample with C_{2v} symmetry with its polar axis along y. MD transition moments are non-zero in this case. In panel **b**, the polar distortion is set to zero so that the sample possesses D_{2h} symmetry with vanishing MD transition moments. The CD response for this case is zero.

Apparent CD effect for dunes along azimuths ± 45 *degrees.*

Further calculations for the asymmetric dune morphology are given in **Figure S2-10** for the same geometry as depicted in **Figure S2-** ***6*** panel **a.** There we show the peak CD response for fixed azimuth angle ϕ = - 45 degrees (panel **a**), ϕ = +45 degrees (panel **b**), plotted versus refraction angle θ_{mat}. Example CD spectra are given for azimuth angle ϕ = -45 degrees (panel **c**), ϕ = +45 degrees (panel **d**) at various fixed values of refraction angle θ_{mat}. The response is antisymmetric with respect the sample flipping or direction of light incidence and is due primarily to apparent CD as described by Salij *et al*., Ref. [1]. The plots also illustrate that on or near the $\pm$45 degree azimuths, although the response is not symmetric with refraction angle, the sign of the peak CD response is the same for positive and negative refraction angle θ_{mat}. As a result, the apparent CD signature will not cancel for ridge structures as discussed in the next section.

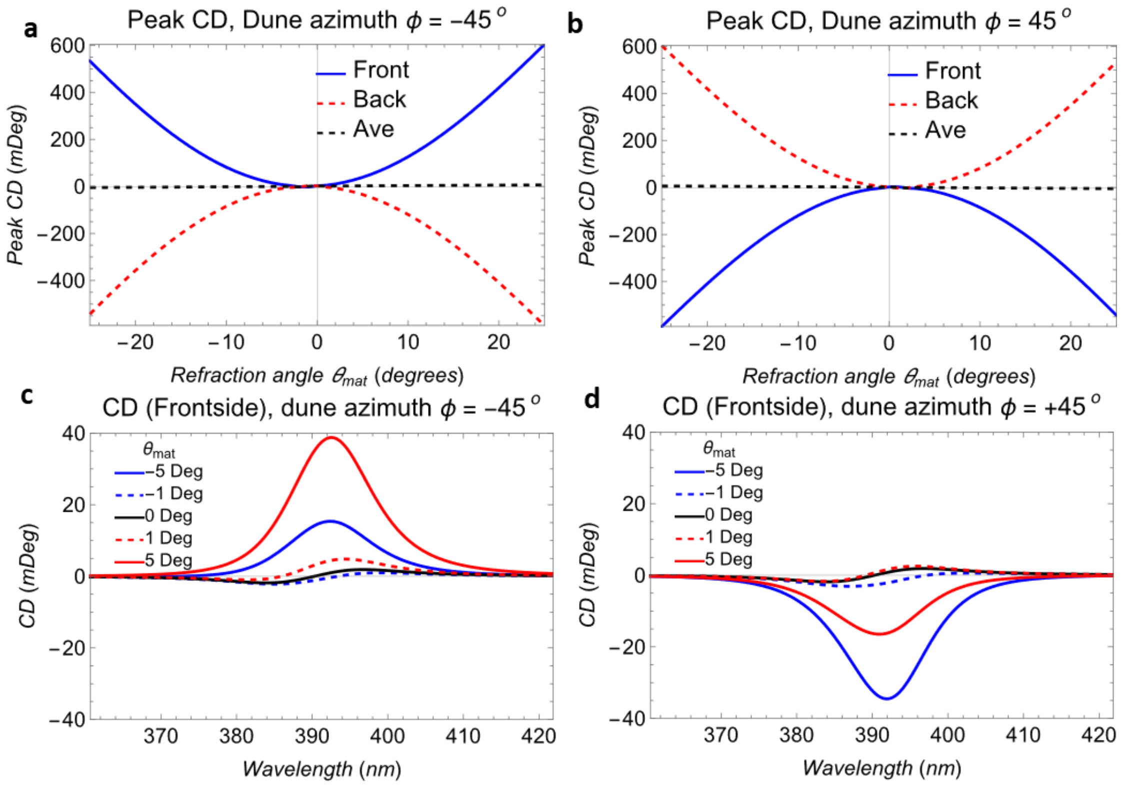

Figure S2- 10. Apparent CD response for dunes at fixed azimuths ϕ = ±45 degrees.
The assumed geometry and material parameters are the same as in **Figure S2- 6**. The peak CD response for fixed azimuth angle ϕ = -45 degrees is shown in panel **a**, and at azimuth angle ϕ = +45 degrees in panel **b**, plotted versus refraction angle θ_{mat}. CD spectra are given for angle ϕ = -45 degrees (panel **c**), ϕ = +45 degrees (panel **d**) at various fixed values of refraction angle θ_{mat}.

S2-4-2-2. Refraction effects: Ridge morphology.

Our discussion of refraction effects associated with the dune structures was predicated on the fact that the dune structures, with their structural asymmetry transverse to the direction of the dune crest alignment, give rise to a strong refraction asymmetry. Consequently dune structures aligned along a given azimuth will have refraction angles θ_{mat} predominantly positive or negative, but not both. In **Figure S2-11** we illustrate refraction effects for the ridge morphology where this transverse asymmetry is minimal. For these calculations, in order to illustrate the antisymmetric response of the apparent CD effect with respect to reversal of incident light direction, we simulate a ridge structure which is symmetric from top to bottom (geometry depicted in **Figure S2-11** panel **a**). Although this top-to-bottom symmetry seems unlikely *a priori*, there is evidence for such a structure in the form of the modelling results of Sec. **S2-4-3-1. Ridge birefringence effects with refraction**., in conjunction with measured results for films processed in DMF at 110C and displayed in **Figure 4**. Calculations in **Figure S2-11** show that the spectral signature is generally monosignate and antisymmetric with respect to sample flipping as expected (panel **c**). The maximal CD response occurs when the ridge azimuth is rotated ± 45 or ± 135 degrees from crystallographic direction $\boldsymbol{a_1}$ (panel **b**), with apparent CD contributions vanishing at azimuths of ± 90 degrees, as expected from **Table S4**, panel **d**. Unlike the dune structures, the norm of the peak CD response for ridges is symmetric for positive and negative values of the dune azimuth angle ϕ; however the response reverses sign with sign reversal of ϕ, vanishing at $\phi = 0$.

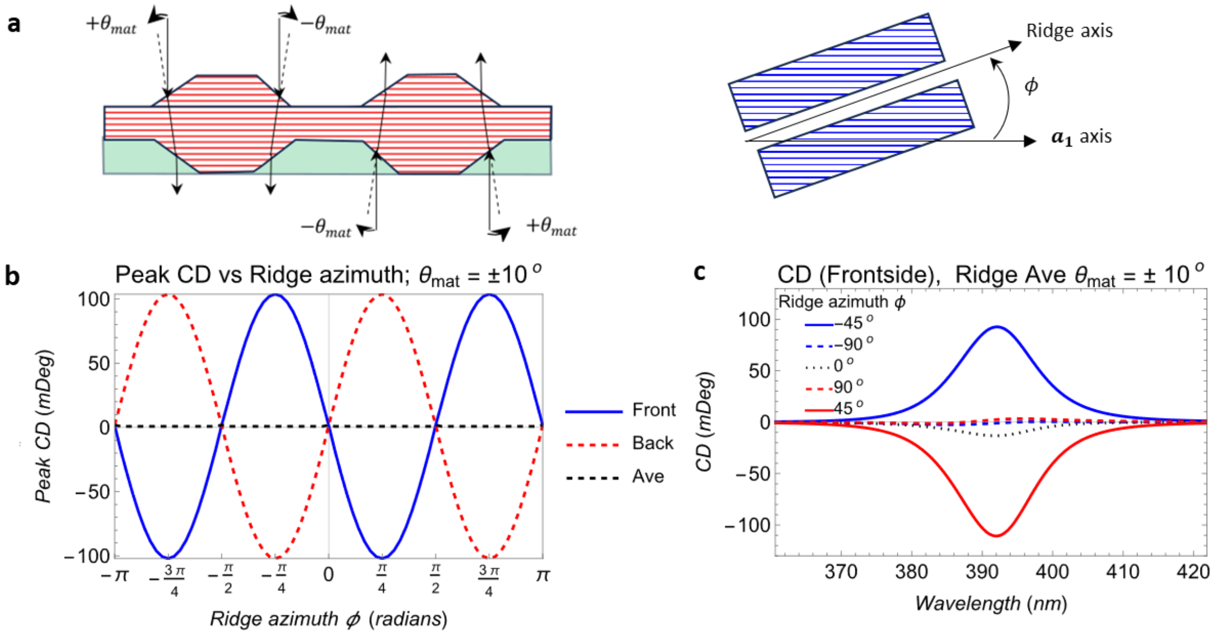

Figure S2- 11. Apparent CD response for symmetric ridges.
The assumed geometry is shown in panel **a**: The ridge structure is taken to be symmetric from top to bottom in order to demonstrate the antisymmetric response with respect to sample flipping. Horizontal red lines depict inorganic layers parallel to the substrate while solid green depicts low index amorphous material. Panel **b** shows peak CD versus azimuth angle ϕ for fixed θ_{mat}=± 10 degrees in equal proportion reflecting the ridge symmetry transverse to the ridge top axis. Panel **c** shows CD spectra for front side illumination at various azimuth angles. The maximum CD response occurs at azimuth angles $\phi = \pm 45$ or ± 135 degrees from crystallographic direction $\boldsymbol{a_1}$. Material parameters are the same as in **Figure S2- 1** for *S*-NPB.

A geometry that may be more realistic for ridge structures, particularly those processed at higher temperatures, is the one shown in **Figure S2-12**, panel **a**, which is assumed to have a flat bottom side morphology. In this case, apparent CD effects vanish when measured from the back side but are identical to those plotted in **Figure S2-11** for front side illumination.

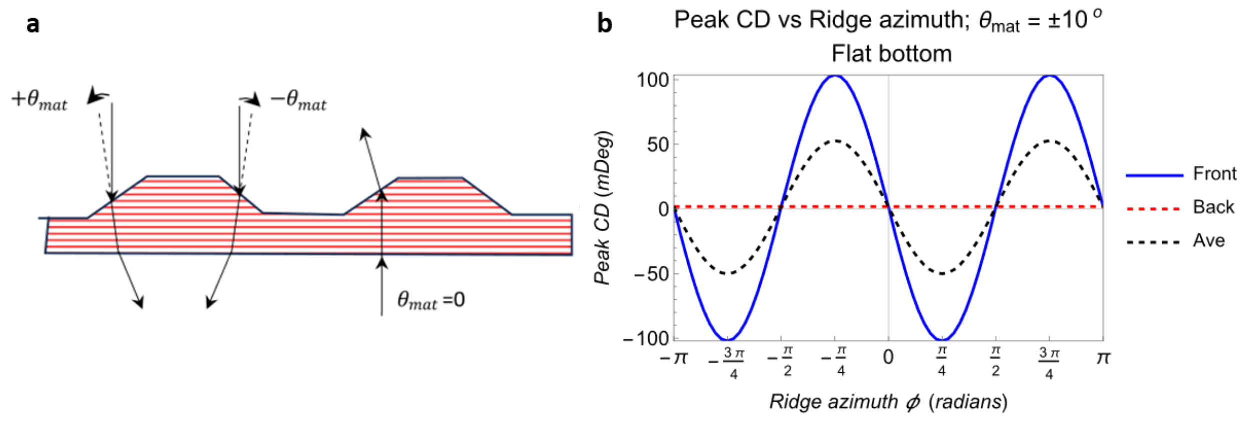

Figure S2- 12. Apparent CD response for ridged films with flat backside.
The assumed geometry is shown in panel **a**: The ridge structure is taken to be symmetric from top to bottom in order to demonstrate the antisymmetry with respect to sample flipping. Horizontal red lines depict inorganic layers parallel to the substrate. Panel **b** shows peak CD versus azimuth angle ϕ for fixed θ_{mat}=$\pm$10 degrees in equal proportion reflecting the ridge symmetry transverse to the ridge top axis. Panel **c** shows CD spectra for front side illumination at various azimuth angles. The maximum CD response occurs at azimuth angles $\phi = \pm 45$ or ± 135 degrees from crystallographic direction $\boldsymbol{a_1}$. Material parameters are the same as in **Figure S2- 1** for *S*-NPB.

Review of main text **Figure 4** shows, however, that very commonly, the backside CD response is monosignate and stronger than the front-side CD response.

Consequently, we must conclude that either the back (substrate) side morphology is often more textured than the front side, which seems unlikely, or, that other effects play a significant role. Motivated by this conclusion, in the next section we consider the possible role of fixed birefringence due, for example, to strain in the ridge structures.

S2-4-3. Ridge birefringence effects.

In addition to refraction effects, the textured morphology of ridges (and dunes) should give rise to birefringence between the ridge parallel and transverse directions. There is some evidence that this is the case in the polarized microscopy images in **Figure S8**, 140°C, where we note that contrast is minimized for ridges parallel to the polarizer and maximized for other ridge orientations, and by the images in **Figure S1**, panel **a**, where the Maltese cross-like pattern indicates an aligned radial axis of growth, as discussed in the main text. This indicates birefringence with major axes aligned to direction of ridges.

Figure S2- 13 shows a simplified cartoon of ridge structures aligned at an azimuth angle ϕ. In the plane transverse to the ridge, the ridge morphology may give rise to relative birefringence $\delta = 2(n_{\parallel} - n_{\perp})/(n_{\parallel} + n_{\perp})$ for light polarized parallel to the ridge versus perpendicular to it. This could be caused by strain or dielectric

screening effects for instance. The birefringence could be confined to the top textured portion of the ridge, **Figure S2-13** panel **b**, or extend through the full thickness of the film, **Figure S2-13** panel **c**.

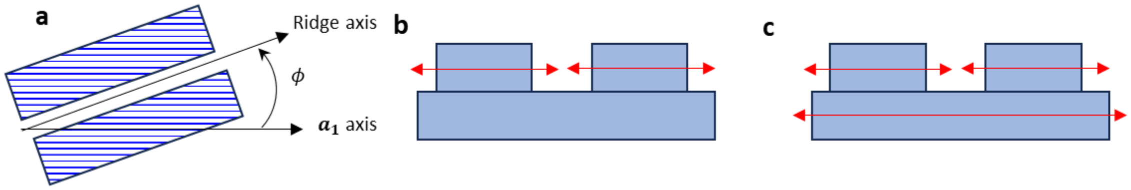

Figure S2- 13. Cartoon depicting birefringence aligned to ridges.
Panel **a**: Ridge top view with the ridge aligned along an azimuth at angle ϕ from the crystallographic direction $\boldsymbol{a_1}$. Panel **b** and **c** respectively depict possible scenarios in which the birefringence is confined to topmost textured portion of the ridge or extends through entire thickness of the film.

Such birefringence effects connected to the ridge morphology can be added into the dielectric model of Eq. S1 by changing the background dielectric portion from an isotropic tensor to one with birefringence. The modified expression for the dielectric tensor, with birefringence associated with a ridge is,

$$\begin{aligned}\boldsymbol{\epsilon}(E) = \epsilon_\infty \Bigg\{ & \mathbf{1} + \boldsymbol{\delta} \\ & + \Delta_{LT} \sum_n \mathcal{L}(E, E_n) \left(\tilde{\boldsymbol{p}}_n{}^* \otimes \tilde{\boldsymbol{p}}_n + h_m \tilde{\boldsymbol{p}}_n{}^* \otimes (\tilde{\boldsymbol{m}}_n \times \hat{k}) \right. \\ & \left. + h_m (\tilde{\boldsymbol{m}}_n \times \hat{k})^* \otimes \tilde{\boldsymbol{p}}_n \right) \Bigg\}, \end{aligned} \tag{S4}$$

where the matrix $\boldsymbol{\delta}$ reflects the relative birefringence between the ridge azimuth and the transverse direction. For ridges whose azimuth is aligned to the x direction (azimuth of 0 degrees in **Figure S2-13**), it has the form:

$$\boldsymbol{\delta} = \begin{pmatrix} \delta & 0 & 0 \\ 0 & -\delta & 0 \\ 0 & 0 & 0 \end{pmatrix}, \tag{S5}$$

For ridges aligned to an arbitrary azimuth, the dielectric tensor can then be found by a simple coordinate transformation. Since this ridge-associated birefringence is, in general misaligned to the exciton linear dichroism, the existence of such ridge birefringence should generate apparent CD which is monosignate and antisymmetric with respect to sample flipping.

The CD response for such birefringence connected to the ridge morphology is depicted in **Figure S2- 14** for films with ridge-associated birefringence that extends through the entire film.

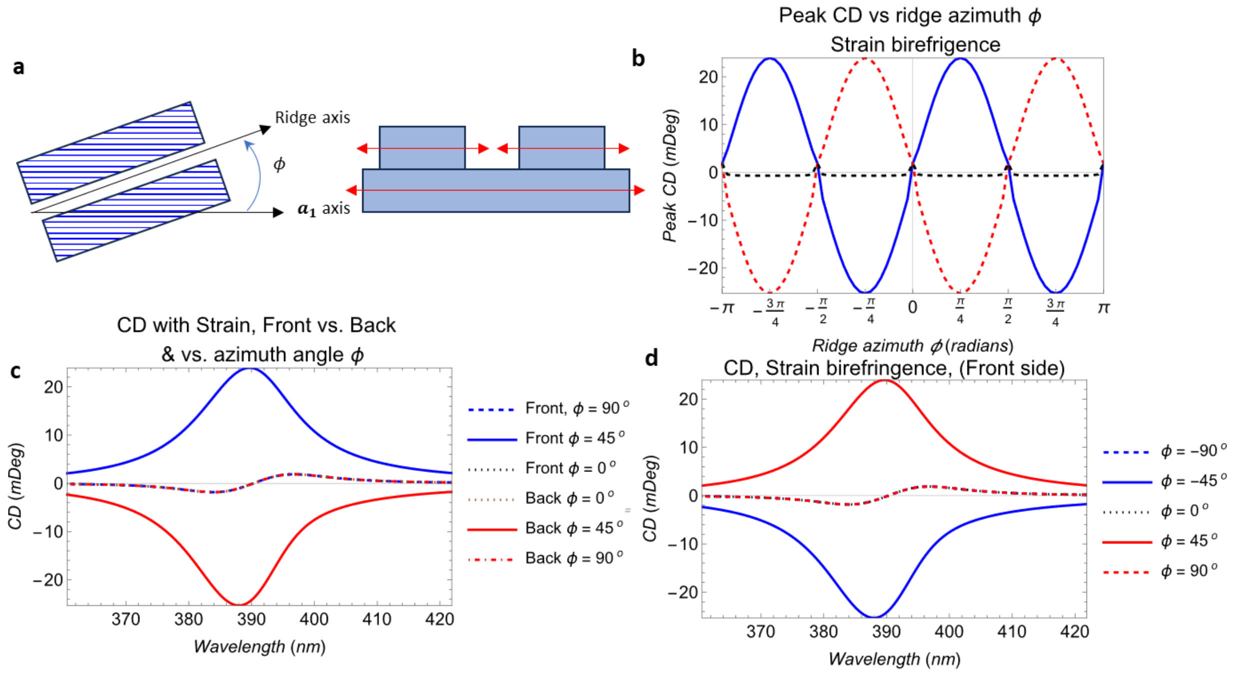

Figure S2- 14. Apparent CD response for ridged films with uniform birefringence (no refraction). The assumed geometry is shown in panel **a**: The ridge structure is taken to have birefringence extending through the entire film thickness, and aligned to the ridge azimuth. Panel **b** shows peak CD versus ridge azimuth angle ϕ for relative birefringence δ =0.001 for front and back side illumination. Panel **c** compares CD spectra for front side versus backside illumination at azimuth angles ϕ =0, 45, and 90 degrees, demonstrating antisymmetric response at 45 degrees and vanishing antisymmetric response at azimuths 0 and 90 degrees. Panel **d** compares CD spectra for front side illumination at azimuth angles ϕ =0, $\pm$45, and $\pm$90 degrees. Note that the spectra at azimuth +45 degrees and -45 degrees have opposite polarity with a spectral shift between the peaks of the two CD spectra. Material parameters are the same as in **Figure S2- 1** for *S*-NPB.

The maximum CD response occurs at ridge azimuth angles $\phi = \pm 45$ or ± 135 degrees from crystallographic direction $\boldsymbol{a_1}$ which is antisymmetric with respect to light incidence direction. The CD response has a vanishing antisymmetric component at azimuths 0 and 90 degrees. The response flips polarity between ridge azimuths +45 and -45 degrees, with a spectral shift between the peaks of the +45 and -45 degree azimuth CD spectra. Most importantly, the ridge-aligned birefringence produces large, monosignate apparent-CD response when measured from the film backside (substrate side). As a result, this model can explain those spectra in main text **Figure 4** which exhibit a large monosignate backside CD response; such spectral features are difficult to explain in terms of the refraction model of **Section S2-4-2**. This is because that the substrate side of the films should presumably be relatively flat in comparison with the top/front side and therefore to not exhibit strong refraction effects.

In **Figure S2- 15** we repeat the ridge-aligned birefringence calculation now assuming that the birefringence is confined to the top-most textured portion of the ridge (panel **a**). In this case the CD response from the top side and the bottom substrate side are non-symmetric, but are both monosignate; and the CD spectra measured from the front and the back side have opposite polarity. As in **Figure S2-14**, the apparent CD response flips polarity between ridge azimuths +45 and -45 degrees, with a spectral shift between the peaks of +45 and -45 degrees azimuth CD spectra.

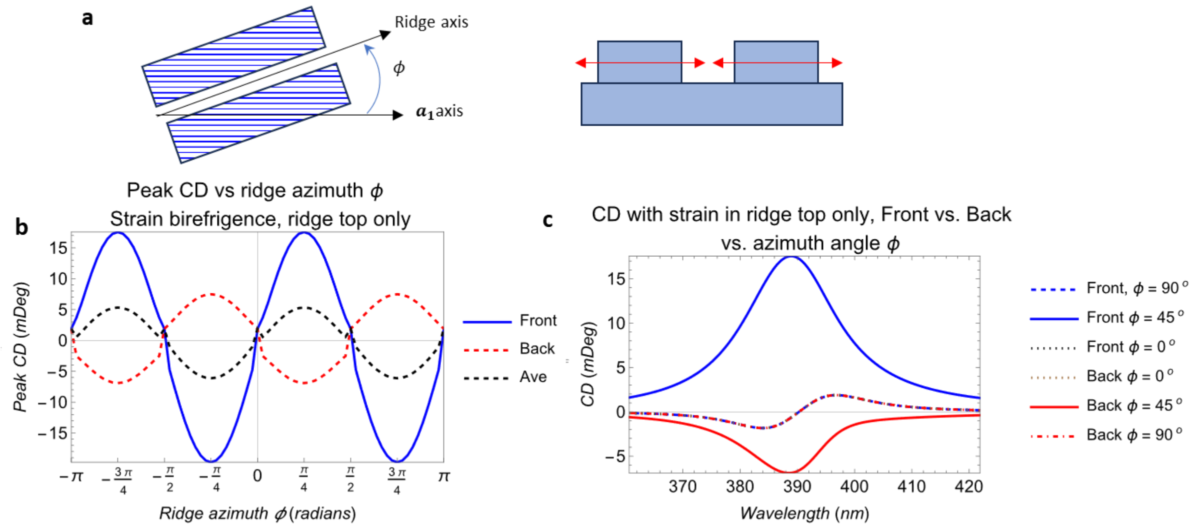

Figure S2- 15. Apparent CD response for ridged films with birefringence in the ridge top (no refraction). The assumed geometry is shown in panel **a**: The ridge structure is taken to have birefringence is confined to topmost textured portion of the ridge extending half way through the film and aligned to the ridge azimuth. Panel **b** shows peak CD versus ridge azimuth angle ϕ for relative birefringence δ =0.001, for both front and back side illumination. Panel **c** compares CD spectra for front side versus backside illumination at azimuth angles ϕ =0, 45, and 90 degrees, demonstrating non-symmetric response at 45 degrees and vanishing antisymmetric response at azimuths 0 and 90 degrees. Material parameters are the same as in **Figure S2- 1** for *S*-NPB.

S2-4-3-1. Ridge birefringence effects with refraction.

The ridge-aligned birefringence effects just described would be expected to co-exist with the sidewall refraction effects discussed in **Section S2-4-2**. An interesting consequence can emerge if both effects are simultaneously present. Comparing the response of the sidewall refraction effect for ridges as a function of ridge azimuth shown in **Figure S2- 11** and **Figure S2- 12** with the ridge azimuth dependence of the CD response associated with ridge birefringence, **Figure S2-15**, we see that the refraction and the strain effects produce responses of opposite polarity for the same ridge azimuth. Moreover, the peak CD from the two effects are spectrally shifted from one another. As a result, when both effects are present, a bisignate apparent-CD response can be observed.

We show this in **Figure S2-16** for a symmetric ridge structure like the one modelled in **Figure S2- 11** but now including the effect of ridge-birefringence (geometry shown in **Figure S2- 16** panel **a**). Panel **b** shows the front-side CD spectral response for ridge azimuth angle ϕ = 45 degrees for relative birefringence δ =0.002 (solid blue line), and separately, the CD response for refraction assuming refraction angle θ_{mat}=±7.5 degrees (dashed red line). The two responses are of opposite polarity and are slightly shifted spectrally. As a result, when both effects are present, the CD spectrum (black solid line) is bisignate. Panel **c** compares CD spectra for front side versus backside illumination at azimuth angle ϕ = 45 degrees, demonstrating antisymmetric bisignate apparent CD response. This scenario may well explain anomalous spectra such as the one measured for films processed at 110C and displayed in **Figure 4** which is bisignate yet antisymmetric with respect to front- versus back -side illumination.

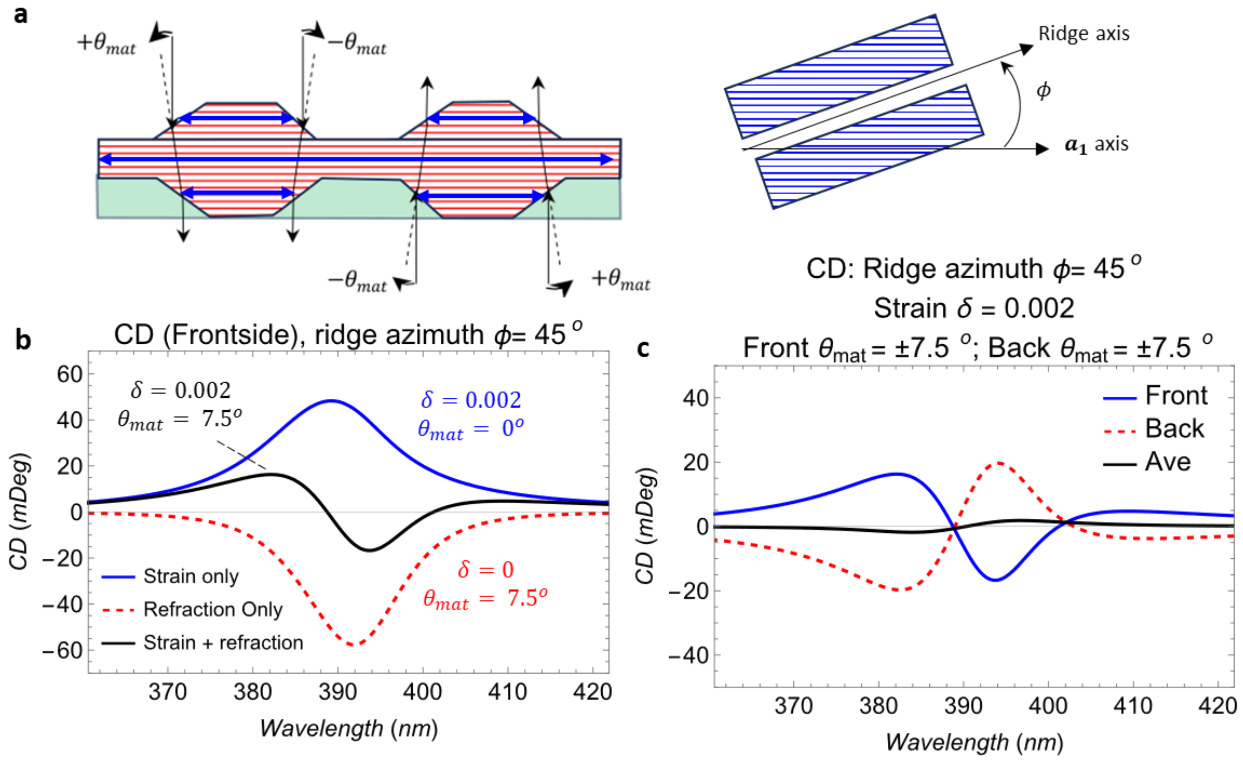

Figure S2- 16. Apparent CD response for ridged films with birefringence and sidewall refraction. The assumed geometry is shown in panel **a**: The ridge structure is taken to be symmetric from top to bottom and to possess uniform ridge-aligned birefringence through the entire film thickness and aligned to the ridge azimuth. Panel **b** shows the front-side CD spectral response for ridge azimuth angle $\phi = 45$ degrees for relative birefringence δ =0.002 (solid blue line), and separately, the CD response for refraction assuming refraction angle θ_{mat}=±7.5 degrees (dashed red line). The two responses are of opposite polarity and are slightly shifted. As a result, when both effects are present, the CD spectrum (black solid line) is bisignate. Panel **c** compares CD spectra for front side versus backside illumination at azimuth angle ϕ = 45 degrees, demonstrating antisymmetric bisignate apparent CD response. Material parameters are the same as in **Figure S2- 1** for *S*-NPB.

References

(1) Salij, A.; Goldsmith, R. H.; Tempelaar, R. Theory of Apparent Circular Dichroism Reveals the Origin of Inverted and Noninverted Chiroptical Response under Sample Flipping. *J. Am. Chem. Soc.* **2021**, *143* (51), 21519–21531. https://doi.org/10.1021/jacs.1c06752.

(2) Jana, M. K.; Song, R.; Liu, H.; Khanal, D. R.; Janke, S. M.; Zhao, R.; Liu, C.; Valy Vardeny, Z.; Blum, V.; Mitzi, D. B. Organic-to-Inorganic Structural Chirality Transfer in a 2D Hybrid Perovskite and Impact on Rashba-Dresselhaus Spin-Orbit Coupling. *Nat Commun* **2020**, *11* (1), 4699. https://doi.org/10.1038/s41467-020-18485-7.

(3) Jana, M. K.; Song, R.; Xie, Y.; Zhao, R.; Sercel, P. C.; Blum, V.; Mitzi, D. B. Structural Descriptor for Enhanced Spin-Splitting in 2D Hybrid Perovskites. *Nature Communications* **2021**, *12* (1), 4982. https://doi.org/10.1038/s41467-021-25149-7.

(4) Sercel, P. C.; Hautzinger, M. P.; Song, R.; Blum, V.; Beard, M. C. Optical Activity of Chiral Excitons. *Advance Materials* **2025**. https://doi.org/10.1002/adma.202415901.

(5) Tinkham, M. *Group Theory and Quantum Mechanics*; Group theory and quantum mechanics; Dover Publications: Mineola, N.Y., 2003.

(6) Clark Jones, R. A New Calculus for the Treatment of Optical Systems. IV. *J. Opt. Soc. Am.* **1942**, *32* (8), 486–493. https://doi.org/10.1364/JOSA.32.000486.
(7) Vernon, R. J.; Huggins, B. D. Extension of the Jones Matrix Formalism to Reflection Problems and Magnetic Materials. *J. Opt. Soc. Am.* **1980**, *70* (11), 1364–1370. https://doi.org/10.1364/JOSA.70.001364.
(8) Agranovich, V.; Ginzburg, V. *Crystal Optics with Spatial Dispersion, and Excitons*; Springer Series in Solid-State Sciences; Springer Berlin, Heidelberg, 2014.
(9) Siegman, A. E. Orthogonality Properties of Optical Resonator Eigenmodes. *Optics Communications* **1979**, *31* (3), 369–373. https://doi.org/10.1016/0030-4018(79)90217-7.
(10) Anthony E. Siegman. Eigenmodes in Nonnormal Optical Systems; 2001; Vol. 4436, pp 1–15. https://doi.org/10.1117/12.451291.
(11) Li, S.; Xu, X.; Kocoj, C. A.; Zhou, C.; Li, Y.; Chen, D.; Bennett, J. A.; Liu, S.; Quan, L.; Sarker, S.; Liu, M.; Qiu, D. Y.; Guo, P. Large Exchange-Driven Intrinsic Circular Dichroism of a Chiral 2D Hybrid Perovskite. *Nature Communications* **2024**, *15* (1), 2573. https://doi.org/10.1038/s41467-024-46851-2.
(12) Sercel, P. C.; Vardeny, Z. V.; Efros, A. L. Circular Dichroism in Non-Chiral Metal Halide Perovskites. *Nanoscale* **2020**, *12* (35), 18067–18078. https://doi.org/10.1039/D0NR05232A.
